# Recent Progress on ZnO Nanowires Cold Cathode and Its Applications

**DOI:** 10.3390/nano11082150

**Published:** 2021-08-23

**Authors:** Yicong Chen, Shaozhi Deng, Ningsheng Xu, Jun Chen

**Affiliations:** State Key Lab of Optoelectronic Materials and Technologies, Guangdong Province Key Lab of Display Material and Technology, School of Electronics and Information Technology, Sun Yat-sen University, Guangzhou 510275, China; chenyc25@mail.sysu.edu.cn (Y.C.); stsdsz@mail.sysu.edu.cn (S.D.); stsxns@mail.sysu.edu.cn (N.X.)

**Keywords:** ZnO nanowire, field emission, large area vacuum microelectronic application

## Abstract

A cold cathode has many applications in high frequency and high power electronic devices, X-ray source, vacuum microelectronic devices and vacuum nanoelectronic devices. After decades of exploration on the cold cathode materials, ZnO nanowire has been regarded as one of the most promising candidates, in particular for large area field emitter arrays (FEAs). Numerous works on the fundamental field emission properties of ZnO nanowire, as well as demonstrations of varieties of large area vacuum microelectronic applications, have been reported. Moreover, techniques such as modifying the geometrical structure, surface decoration and element doping were also proposed for optimizing the field emissions. This paper aims to provide a comprehensive review on recent progress on the ZnO nanowire cold cathode and its applications. We will begin with a brief introduction on the synthesis methods and discuss their advantages/disadvantages for cold cathode applications. After that, the field emission properties, mechanism and optimization will be introduced in detail. Then, the development for applications of large-area ZnO nanowire FEAs will also be covered. Finally, some future perspectives are provided.

## 1. Introduction

**Figure 1:**
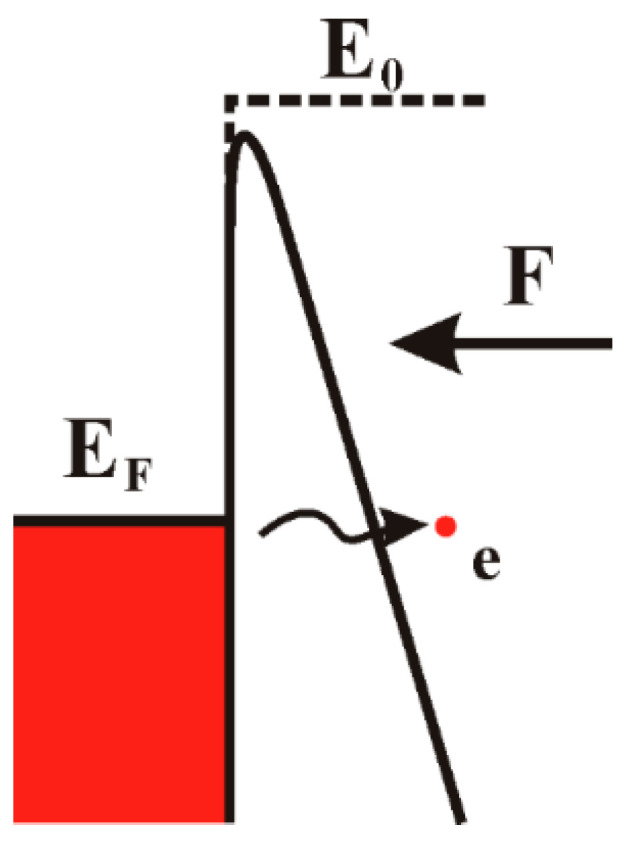
Energy band diagram of field electron emission from metal. E_F_ and E_0_ are respectively the Fermi level and vacuum level of metal cathode under zero field. When a field F is applied on the surface, the vacuum barrier will bend. Electrons can tunnel into the vacuum if the barrier width is in several nanometers scale.

When a strong field is applied on a cathode surface, its vacuum barrier will bend. If the barrier width for an electron is as narrow as several nanometers, there is a considerable probability the electrons will tunnel into the vacuum (Figure 1). This process is referred to as field electron emission. Field electron emission is the basic principle of a cold cathode. Compared to a traditional thermionic cathode, a cold cathode has many advantages, such as fast response time, low power consumption, narrow energy spread, compact size, high brightness, etc. One of the most attractive features of a cold cathode is probably its capability in miniaturized and addressable devices, which creates the concept of field emitter arrays (FEAs) that has potential applications in field emission display (FED) [1], parallel electron beam (e-beam) lithography [2], flat panel X-ray source [3] and large area photodetectors [4].

The first serious investigation on FEAs can be traced back to the 1960s by Spindt et al. [5], who developed the Spindt-type FEAs that consist of molybdenum tips using micro-fabrication method. After that, silicon tip arrays were also fabricated. Along with the discovery of carbon nanotube (CNT) and other quasi-one-dimensional (Q1D) nanowires, many efforts have been made to investigate the nanotube/nanowire FEAs during the first decade in the 21st century, because of their high aspect ratio and unique properties. Moreover, field emission from two-dimensional layered material FEAs such as graphene was also explored. Although many works have reported excellent field emission properties from individual Q1D field emitter, e.g., LaB_6_ nanowire [6], Mo nanostructure [7], etc., they are still difficult to fabricate in a large area with acceptable uniformity in field emission.

After decades of research, only the Spindt-type FEAs, CNT and ZnO nanowire are still active in the field of addressable FEAs. Compared to the Spindt-type FEAs and CNT, a ZnO nanowire has many advantages, such as stable chemical properties, simple synthesis method, low cost and good compatibility with micro-fabrication technics, which make it promising for large area addressable FEAs. To further improve the field emission properties of ZnO nanowire FEAs, it is necessary to review their recent progress and outline future research trends.

Although there are already some reviews [8,9] for Q1D ZnO nanostructure cold cathodes, they mainly addressed on the synthesis method. This review will focus on the field emission of ZnO nanowire, including the mechanism, optimization technique and large area vacuum microelectronic device applications. Generally, Q1D ZnO nanostructures contain geometries of nanowire, nanorod, nanotetrapod, etc. Since the basic physical properties of ZnO nanostructures, such as energy bandgap and work function, are independent of the geometry, reports on ZnO nanowire as well as other Q1D ZnO nanostructures will be covered in this review.

Finally, future perspectives for the improvement of emission current density and electron beam convergence are provided. We hope this review can be helpful for not only encouraging the people who still engage in this field but also guiding new researchers.

## 2. Synthesis

To date, ZnO nanowires have been synthesized by many methods, such as chemical vapor deposition (CVD), hydrothermal method and thermal oxidation. Considering that the growth mechanisms of these methods have been introduced in detail in many other reviews [10,11,12,13], in the following we will mainly focus on their advantages and disadvantages in the applications of a cold cathode.

### 2.1. Chemical Vapor Deposition

Although ZnO nanowires have been reported to be synthesized by many methods such as thermal evaporation [14], physical vapor deposition [15], and metal organic chemical vapor deposition [16], they are basically different kinds of CVD. This is because all these methods need to transfer the vapor species onto the substrate, then let them react under high temperature and produce high-quality nanowires. For simplicity, these kinds of vapor synthesis method are referred to as CVD in this review.

The most outstanding advantage of the CVD growth ZnO nanowires is probably their high crystalline quality, which results in a high field emission current from individual nanowire in many of the reported works (results for nanowires film and individual nanowire can be seen in Table 1 and Table 2). Moreover, the high synthesis temperature allows doping of many materials in ZnO nanowires, e.g., Al [17], In [18], Cu [19], Ge [20], which can help to tune their conductivity and field emission properties.

**Figure 2:**
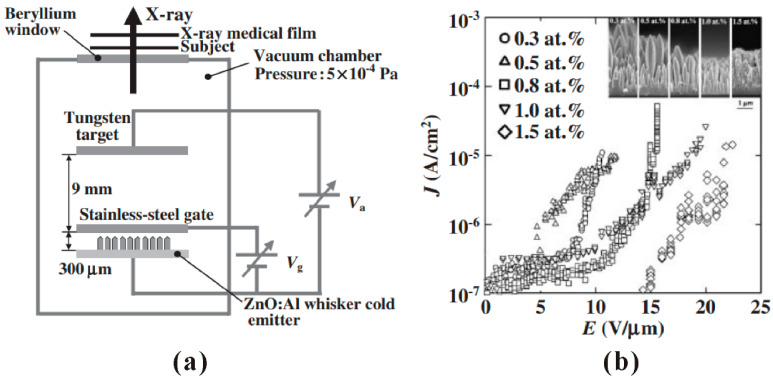
Demonstration of a X-ray source based on chemical vapor deposition (CVD)-synthesized ZnO:Al whiskers. (**a**) Schematic diagram of the discrete device structure. (**b**) Field emission properties of ZnO:Al whiskers with different Al concentration. The inset shows the morphology of the whiskers. Reproduced from [22], with the permission of the Japan Society of Applied Physics, 2008.

However, it is difficult to integrate these high-temperature synthesized ZnO nanowire arrays in a large area gated device using the modern micro-fabrication technique. So far, only S.Y. Li et al. [21] have demonstrated a gated CVD growth ZnO nanowires device on Si substrate. To avoid the compatibility problem, S. Ooki et al. [22] utilized a discrete device structure to fabricate a gated X-ray source based on CVD growth ZnO:Al whiskers, the typical device structure and field emission properties of which can be seen in Figure 2. K. Zheng et al. [23] screen-printed the CVD grown ZnO nanotetrapods on the Ag electrode strips and used a phosphor-coated anode with electrode strips perpendicular to the Ag strips to realize field emission display. Besides, Q. Zhao et al. [24] demonstrated a lateral field emission device using individual ZnO nanowire grown by CVD.

### 2.2. Hydrothermal Method

The hydrothermal method is also referred to as the solution phase synthesis. Its general synthesis process is as follows. Firstly, a thin layer of ZnO will be deposited on a substrate as the seed layer. Then, the substrate is suspended in the nutrient solution and kept at a certain temperature (lower than 200 °C) for a period of time. Usually, the nutrient solution is a mixture of alkaline reagent and Zn^2+^ salt (Zn(NO_3_)_2_, ZnCl_2_, etc.). Finally, the resultant substrate is washed and dried.

**Figure 3:**
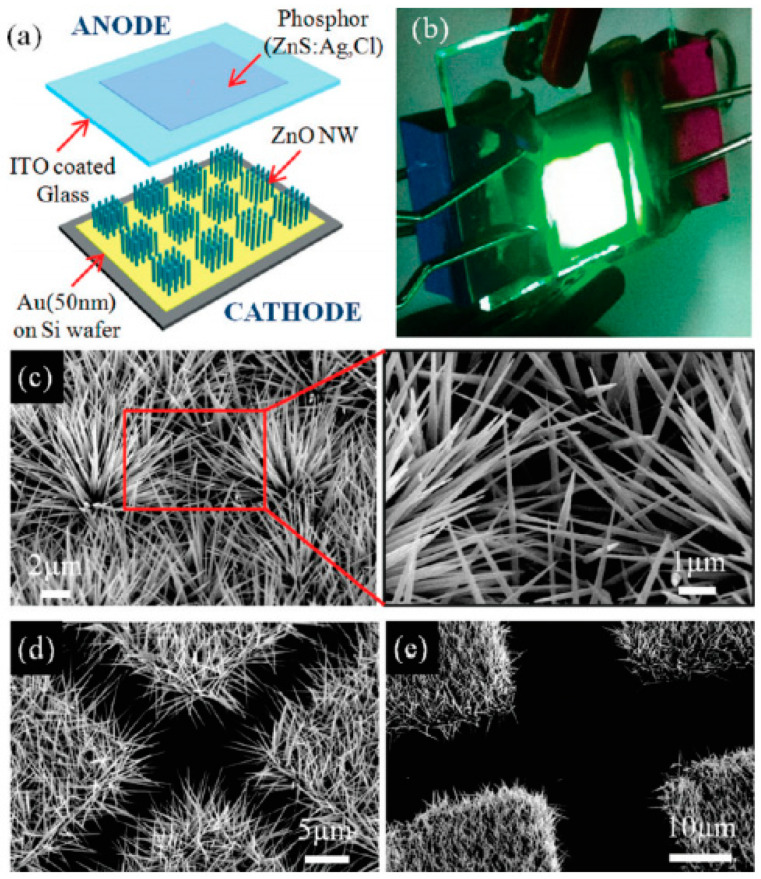
Demonstration of a diode field emission device based on hydrothermal-synthesized ZnO nanowires. (**a**) Schematic diagram and (**b**) optical image of the ZnO nanowires cold cathode. (**c**–**e**) scanning electron microscope (SEM) image of the nanowire arrays with different pattern spaces. Reproduced from [25], with the permission of ACS Publications, 2011.

Compared to the CVD method, the hydrothermal method has advantages of low temperature, low cost and scalability. These make it possible to fabricate ZnO nanowires on various kinds of substrate. As an example, H.W. Kang et al. [25] demonstrated a field emission device using hydrothermal synthesized ZnO nanowire on both inorganic and organic substrate. Typical results for the device and the morphology of the hydrothermal synthesized nanowire are illustrated in Figure 3. Besides, by tuning the seed layer [26,27], nutrient solution [28,29,30,31] and growth time [32], the morphology, density and doping of nanowire can be modified, which offer important ways to optimize their field emission properties.

However, low-temperature synthesized nanowires usually have a poorer crystalline quality, which results in a lower emission current from individual nanowire (results for nanowires film and individual nanowire can be seen in Table 1 and Table 2). Furthermore, although the gated device based on hydrothermal synthesized ZnO nanowires has been demonstrated [33], it is still difficult to integrate them in a field-emission device with addressing capability, which may be attributed to the compatibility problem.

### 2.3. Thermal Oxidation

Thermal oxidation is probably the simplest approach for fabricating a large area ZnO nanowire cold cathode. A typical procedure for the thermal oxidation method can be described as two steps. One is the deposition of Zn thin film, which can be usually carried out by the electron beam evaporation of Zn. The other is heating the sample in furnace at air atmosphere under ~500 °C After cooling down to room temperature, ZnO nanowires can be synthesized.

**Figure 4:**
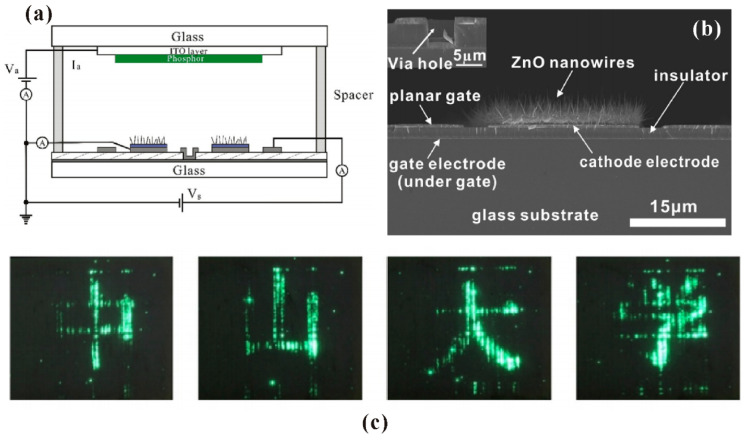
Demonstration of an addressable field emission device based on thermal oxidized ZnO nanowires. (**a**) Schematic diagram and (**b**) cross-sectional SEM image of the device. (**c**) Some Chinese characters displayed by the device. Reproduced from [34], with the permission of Elsevier, 2017.

Since the location of nanowire is defined by the initial Zn thin film, ZnO nanowires can be synthesized according to the desired pattern which can be easily controlled by the standard micro-fabrication process. Moreover, considering that the synthesis temperature of 500 °C is below the melting point of commercial glass, this method enables the synthesis of patterned ZnO nanowires in large area glass substrate. Furthermore, no catalyst and extra gas are needed during the whole synthesis process which reduces the cost. All these features make it promising in preparation of a large-area gated ZnO nanowire cold cathode. As an example, a diagonal 3.5-inch addressable field emission device based on thermal oxidized ZnO nanowires has been demonstrated by L. Zhao et al. [34], the device structure and performance of which are illustrated in Figure 4. Other addressable FE devices [35,36,37] are also reported.

Compared to the ZnO nanowires synthesized by the high-temperature CVD method, the oxidized nanowires usually have a poorer crystalline quality [38]. However, they have a higher maximum field emission current density from individual emitter, which can be seen in Table 2. This can be attributed to the effects of the defects. More details for the mechanisms will be discussed in the next section. A few optimization works on the synthesis process of the thermal oxidation method have been carried out. For example, in most of the reported works, only the nanowire morphology [39] and density [35,40] can be modified by adjusting the stress of the initial Zn thin film. Even though a recent work has realized In doping in oxidized ZnO nanowire [41], the control of doping concentration and other high melting point materials doping still remain challenging.

## 3. Field Emission

Study of the field emission of ZnO nanowires is the key for their applications as cold cathode. In this section, the field-emission properties, mechanism and optimization will be introduced. Considering that the field-emission properties of ZnO nanowires have been investigated for nearly two decades since the pioneering works [42,43,44], people may be more interested in the progress of their field-emission mechanism and optimization techniques. Therefore, this review will give a brief summary on the basic values (such as turn-on field, maximum current density, etc.) for the field-emission properties of ZnO nanowires and mainly focus on the parts of mechanism and optimization.

### 3.1. Field-Emission Properties

Generally, the field-emission properties of cold cathode contain the field-emission current-voltage (I–V) characteristics, field-emission pattern, field-emission energy distribution and emission current stability, which could tell us the information about the turn-on field, maximum current, field-emission spatial distribution, full width at half maximum (FWHM) of emission electron energy and life time of the field emitter. For the ZnO nanowire cold cathode, the reported field-emission measurements have been carried out on not only the nanowires film but also individual nanowire which could help to investigate its intrinsic field-emission properties. Considering that the field-emission properties of the nanowires film is related to both the intrinsic properties of individual nanowire and the average effect of numerous nanowires, e.g., screening effect, they can be totally different from that of individual nanowire. To obtain a better understanding, we classify this content into two parts: one is the result for nanowires film and the other is that for individual nanowire.

#### 3.1.1. Nanowires Film

**Figure 5:**
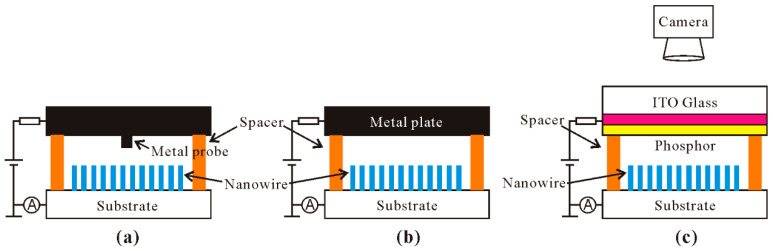
Schematic diagrams for the measurement setup with a metal probe (**a**), metal plate (**b**) and phosphor-coated ITO glass (**c**) as the anode.

In the field-emission measurement of nanowire film, people are mainly concerned with the turn-on field, maximum current density, stability and uniformity, which are reflected in the field-emission I–V characteristics and field-emission pattern. To measure the field-emission I–V characteristics, one can use a metal probe or a parallel metal plate as the anode. To measure the field-emission pattern, a phosphor-coated ITO glass can be used as the anode and a CCD camera is applied for recording the pattern. The corresponding setup can be seen schematically in Figure 5, where the distance between anode and cathode plates is determined by the spacer.

**Figure 6:**
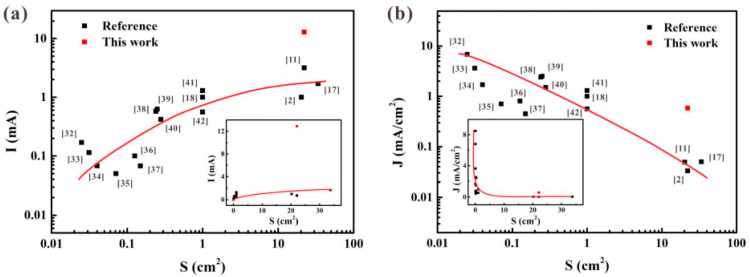
Relationships of field emission current (**a**) and current density (**b**) versus emission area plotted by reported works. The insets are the same results in linear coordinates. Reproduced from [40], with the permission of Elsevier, 2019.

After nearly two decades’ research on the field emission from ZnO nanowires film, it was found that this cold cathode material has a turn-on field as low as ~1 V/μm for obtaining the current density of 10 μA/cm^2^ and a maximum current density in the range of 10–100 mA/cm^2^, and some of the reported results can be seen in Table 1. It is worth pointing out that the emission area for calculation of current density is usually the overlap area between anode and cathode, e.g., when using a metal probe as the anode, the emission area is equal to the probe area, while using a metal plate as the anode, the emission area is equal to the cathode area. For the patterned ZnO nanowires, although the effective emission area is much smaller than the cathode area, their current density is still calculated following the above methods. Under this framework, L. Wang et al. [40] proposed that the emission current density of ZnO nanowires will decrease as the cathode area increases by plotting the reported results as shown in Figure 6. This is understandable due to the screening effect. Since one of the ultimate goals for the development of ZnO nanowires’ cold cathode is the application in large area field emitter arrays, they modified the pattern spacing to overcome this problem and obtained the highest value of current density with a cathode area > 20 cm^2^. Details for the impact of this modification will be introduced below.

**Table 1:** Summary for recent works on the field emission properties from ZnO nanowires film. The turn-on field is defined as the applied field needed to obtain the emission current density of 10 μA/cm^2^. “---” means the corresponding value is not provided in the reference. CVD is short for chemical vapor deposition.

Cathode Materials	Institution	Synthesis Method	Measured Area (cm^2^)	Turn-On Field (V/μm)	Maximum Current Density (mA/cm^2^)
ZnO nanoneedles film [45]	Chinese Academy of Sciences	CVD	0.005	~5.3	0.6
ZnO nanowires film [46]	City University of Hong Kong		~0.008	1.1	30
ZnO nanotips film [17]	Zhejiang University		---	1	~7
Al doped ZnO nanowires film [17]			---	0.5	>1.2
In-doped ZnO nanowires film [18]	Tsinghua University		---	2.4	>1.6
ZnO nanowires on plate structure [47]	Nanjing University of Science and Technology		0.01	4.8	7.8
Ge-doped ZnO nanowires [20]	Guangzhou Maritime University		~0.0013	3.5–4.1	90
ZnO nanoflowers film [48]	Najran University		0.25	~5	~0.075
ZnO nanowires film [49]	Lanzhou University		---	<3.79	~6
Al-doped ZnO nanowires film [31,50]	Northwest University	Hydrothermal method	1	~2.5	0.3–0.55
ZnO nanorods film [51]	Southeast University		1	~2.5	~1
ZnO nanotubes film [52]	Nanyang Technological University		~0.2	~11	~1
ZnO nanowires film [53]	University of Lyon 1		~0.00049	---	50
ZnO nanowires film [54]	Korea Advanced Institute of Science and Technology		~16	2–2.8	>1
Patterned ZnO nanowires [25]			1	1.6–2.45	>1
ZnO nanotapered arrays film [55]	Shanghai Jiao Tong University		0.25	1.5–19.7	>1
ZnO nanowires film [26]	Sun Yat-sen University		~0.008	7.1	~25
ZnO nanorods film [56]	Jadavpur University		~0.008	3.2–7.4	>6
Patterned ZnO nanowires [35,38,39,40]	Sun Yat-sen University	Thermal oxidation	1–36	4.53–7.8	0.35–1.3
Patterned In-doped ZnO nanowires [41]	Sun Yat-sen University		23	7.1	~0.84

**Figure 7:**
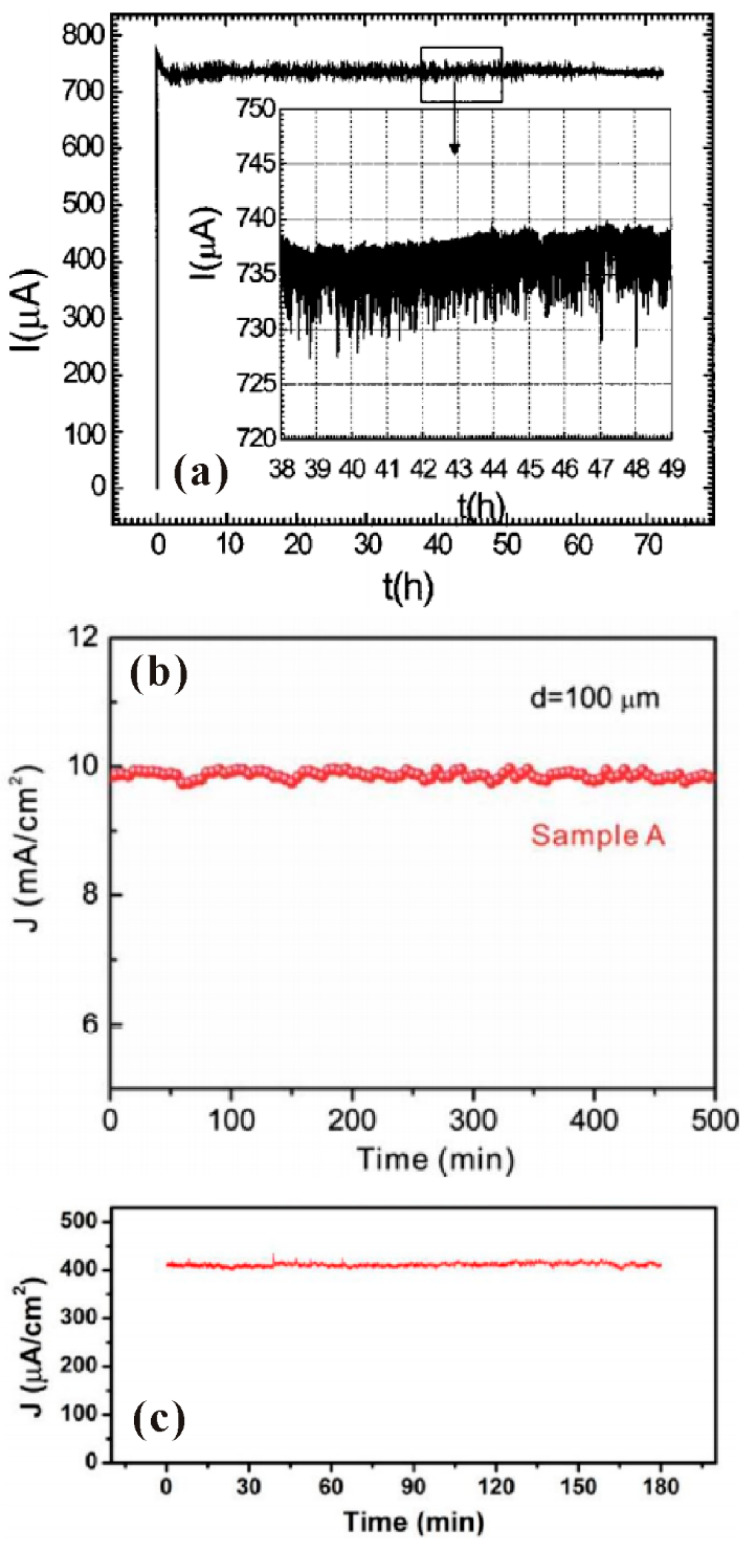
Field emission stability of ZnO nanowires. (**a**) ZnO nanowires painted on Au-Si substrate. Reproduced from [59], with the permission of AIP Publishing, 2004. (**b**) ZnO nanowires grown on Al: ZnO substrate. Reproduced from [46], with the permission of AIP Publishing, 2009. (**c**) ZnO nanowires directly grown from Zn thin film on ITO glass. Reproduced from [38], with the permission of ACS Publications, 2013.

The stability is another important issue for the application of a cold cathode. Although a cascade structure by which the ZnO nanowires are connected with a TFT [57] or MOSFET [58] in series can improve the stability of the device, this review mainly concerns the intrinsic stability of the cathode, which can give a better comparison with other cold cathode materials. So far, it has been found that the fluctuation of emission current of ZnO nanowires film can be as low as 1–2% during the long-term test for over 3 h [38,46,59]. Moreover, a slow increase in the emission current can be observed [40,60]. Generally, a good contact between the nanowire and the substrate is responsible for the high stability. For example, Q.H. Li et al. [59] obtained the current fluctuation of <2% (Figure 7a) from ZnO nanostructures by covering an Au layer between the nanostructure and the Si substrate for improving their contact. Z.H Chen et al. [46] used a Al:ZnO buffer layer to provide effective electrons injection into the ZnO nanowire, which can improve thermal stability during field emission process thus lead to a low current fluctuation of 0.6% (Figure 7b). C.X. Zhao et al. [38] also obtained the current fluctuation of <1% (Figure 7c) from the ZnO nanowires directly grown from the Zn thin film on ITO-coated glass, which can reduce the back-contact resistance between the nanowire and the substrate. The current increase phenomenon is believed to be related to the joule heating effect of the nanowire, which will be introduced in detail in the next part. Although the exact value for the life time of ZnO nanowire is difficult to be deduced from the presented results, stable field emission from ZnO nanowires film for over 70 h has been demonstrated [59].

**Figure 8:**
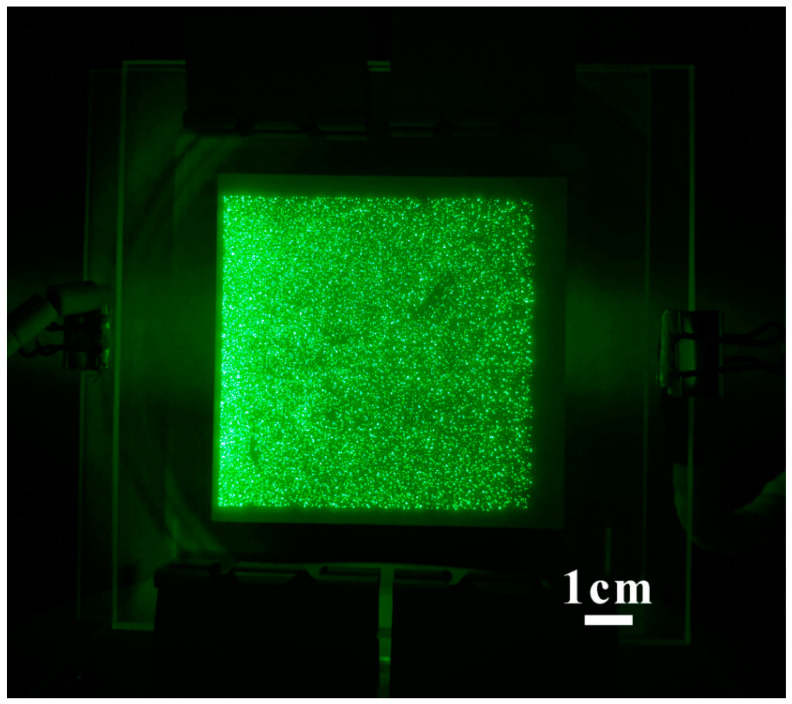
Field emission pattern of a diagonal 4-inch ZnO nanowires array. Reproduced from [61], with the permission of IEEE, 2021.

In addition, uniformity is also a significant factor for evaluating the performance of large-area FEAs. Although an excellent uniformity has been demonstrated in a small size device [25] (usually <1 cm^2^), large area field emitter arrays with high uniformity is still a challenge, which is mainly due to the difficulties in fabrication of large-area uniform ZnO nanowires and elimination of field emission hot spots. As the cathode area increases, more hot spot sites may exist which can easily cause damage to the nanowires. In the present stage, the maximum reported size for ZnO nanowires cold cathode with acceptable uniformity is 4 inches diagonally [61], which were fabricated by the thermal oxidation method in glass substrate and performed after a conditioning process (Figure 8). Further investigation on the field-emission conditioning process, especially for elimination of multi-hot spot sites, is expected.

#### 3.1.2. Individual Nanowire

**Figure 9:**
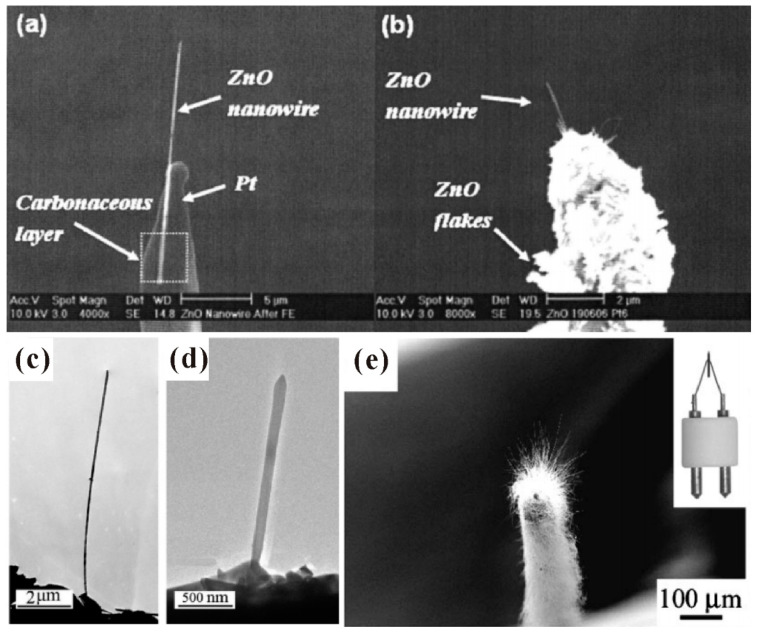
Single ZnO nanowire fabricated on platinum (**a**,**b**) and tungsten (**c**–**e**) probes. In (**a**,**c**,**d**), the nanowires were attached on the probe. While in (**b**,**e**), the nanowires were grown on the probe. (**a**,**b**) Reproduced from [62], with the permission of AIP Publishing, 2008. (**c**,**d**) Reproduced from [63], with the permission of ACS Publications, 2007. (**e**) Reproduced from [64], with the permission of AIP Publishing, 2003.

**Figure 10:**
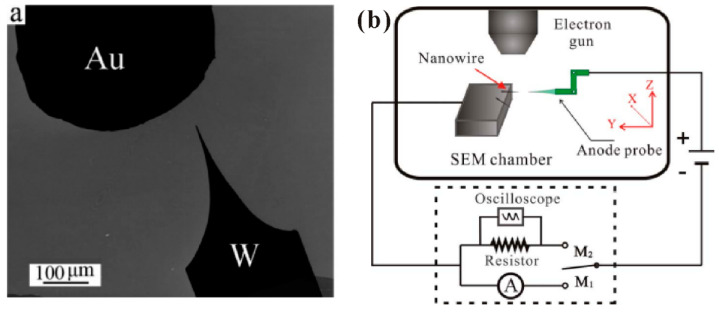
Schematic diagrams of the setup for in situ field-emission measurement in a transmission electron microscope (TEM) (**a**) or SEM (**b**) system. (**a**) Reproduced from [63], with the permission of ACS Publications, 2007. (**b**) Reproduced from [65], with the permission of AIP Publishing, 2009.

In the measurement of an individual nanowire, the maximum emission current, field-emission spatial distribution and field-emission energy distribution are three aspects that researchers are mostly concerned with. Generally, there are two kinds of technique to perform field emission measurement on individual nanowire. One is to fabricate individual nanowire on a metal tip as the cathode, then utilize a phosphor coated plate or faraday cup as the anode. Some SEM or TEM images for a single ZnO nanowire on a platinum or tungsten tip can be seen in Figure 9, which were fabricated by attaching or directly grown methods. This individual nanowire sample can also be measured in a TEM system with a movable sample holder and a fixed gold ball as the anode (Figure 10a). The other is to equip a manipulated anode probe in a SEM system, then move the anode probe near the nanowire tip for in situ electrical and field emission measurements. The schematic diagram for the corresponding setup can be seen in Figure 10b.

Table 2 lists the reported works for an individual ZnO nanowire, which indicates that the maximum current can be as high as 5 μA. One may also find that the CVD-grown ZnO nanowire has a larger maximum current ranging from 0.9 to 5 μA than those synthesized by hydrothermal method or thermal oxidation. However, by calculating the corresponding current density with the assumption that the emission area is equal to the cross-sectional area of the nanowire, it can be seen that these nanowires have a similar current density in the magnitude of ~10^4^ A/cm^2^. For the thermal oxidized ZnO nanowire, its maximum current density can even reach 10^6^ A/cm^2^. Therefore, the large maximum current for the CVD-grown ZnO nanowire originates from its larger size, which can carry more current.

**Table 2:** Summary of field emission properties from individual ZnO nanowire. CVD is short for chemical vapor deposition.

Cathode Materials	Synthesis Method	Length (μm)	Diameter (nm)	Maximum Current (μA)	Current Density (A/cm^2^)
In-doped ZnO nanowire [63]	CVD	~1.7	100	~1.5	1.9 × 10^4^
ZnO nanowire [63]	---	~4.3	100	~0.75	9.5 × 10^3^
ZnO nanowire [66]	CVD	~6	~100	~5	6.4 × 10^4^
Agave-like ZnO nanowire [67]	CVD	30	180	~1.6	6.3 × 10^3^
Agave-like ZnO nanowire [65]	CVD	30	150	~0.9	5.1 × 10^3^
ZnO nanowire [27]	Hydrothermal method	0.67	40	~0.017	1.4 × 10^3^
ZnO nanowire [62]	Thermal oxidation	~2	~10	>1	1.3 × 10^6^
ZnO nanowire [68]	Thermal oxidation	1	26	~0.4	7.5 × 10^4^

**Figure 11:**
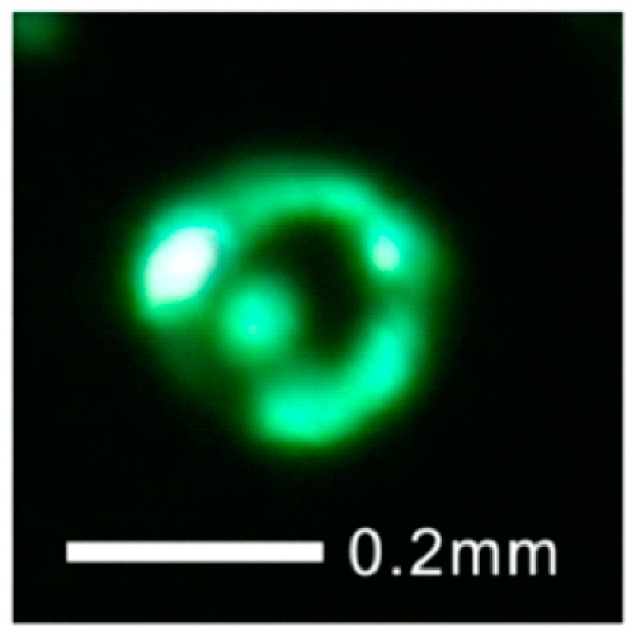
Field-emission ring pattern of individual ZnO nanowire. Reproduced from [70], with the permission of IOP Publishing, 2014.

**Figure 12:**
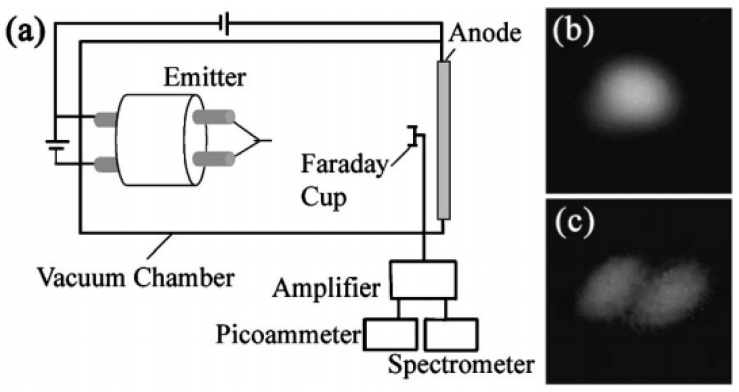
(**a**) The measurement setup for angular intensity of individual ZnO nanowire. (**b**,**c**) Typical field-emission pattern of the nanowire. Reproduced from [64], with the permission of AIP Publishing, 2003.

The field-emission spatial distribution has important influences on the convergence of the electron beam and its brightness. In the works of X. Zhang et al. [69] and Y.C. Chen et al. [70], the ring-shaped field emission pattern as shown in Figure 11 has been observed from an individual ZnO nanowire. Besides, L. Dong et al. [64] measured the angular intensity of <0.1 mA/sr by using the setup shown in Figure 12a, which the field emission patterns can be seen in Figure 12b,c. All these results indicate that the presented ZnO nanowire may have a wide range of emission angle at its tip, which makes it unsuitable for highly converged electron source.

**Figure 13:**
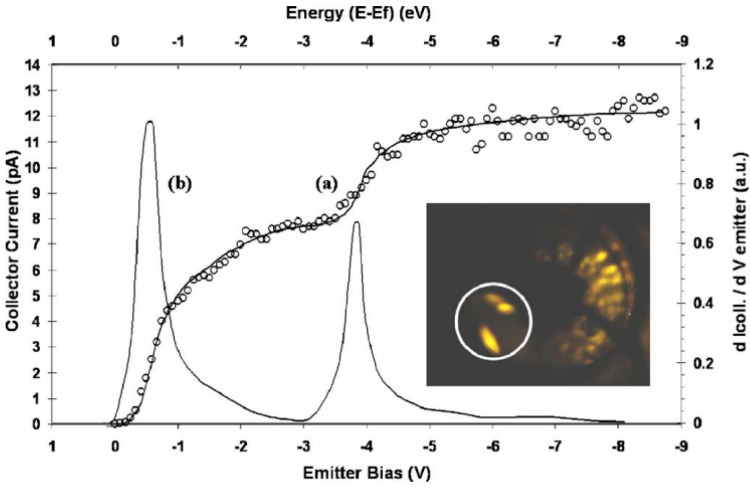
Field emission energy spectra from a ZnO taprapod emitter. (**a**) Collector current versus emitter bias characteristic. (**b**) Differentiation of (**a**). Inset is the field emission pattern. Reproduced from [71], with the permission of AIP Publishing, 2007.

So far, only A.A. Al-Tabbakh et al. [71] has measured the field emission energy distribution from an individual ZnO nanostructure. Figure 13 shows the result carried out by a retarding energy analyzer. They found that the emission electrons come from both the conduction and valence bands, which results in two peaks in the energy spectra. Although the FWHM of each peak can be smaller than 0.5 eV, the overall energy spread is larger than 3.3 eV, which makes it difficult to be converged in the application of a high-resolution electron beam. More research on this aspect is required for comprehensive knowledge of its emission mechanism.

**Figure 14:**
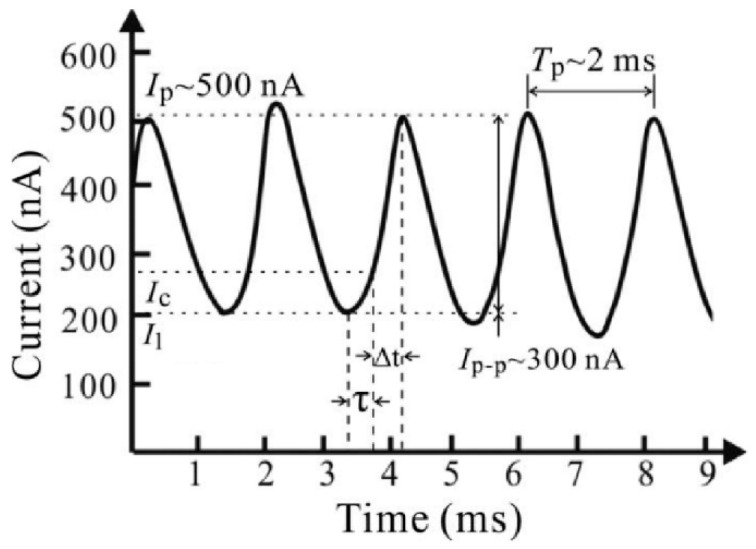
The sine-form oscillating current from field emission of an individual ZnO nanowire. Reproduced from [65], with the permission of AIP Publishing, 2009.

Interesting phenomenon such as the emission current oscillating (Figure 14) was also reported in the field emission of individual ZnO nanowire by Z. Xiao et al. [65], which they attributed to the self-heating induced melting process at the nanowire tip. Details for the self-heating of a ZnO nanowire will be introduced in the mechanism part (Section 3.2).

### 3.2. Mechanism

Before we introduce the development of the field-emission mechanism from a ZnO nanowire, it is necessary to briefly review the Fowler–Nordheim (FN) theory, which has successfully explained the field emission from metal at low temperature since 1928 [72]. The FN theory gave a simplified exponential relationship between the emission current density *J* and the surface field *F* as follows,

(1) $$J=\frac{AF^{2}}{\phi \;}\exp\left(\frac{-B\phi^{3/2}}{F}\right)$$

where *φ* is the work function of the cathode, *A* = 1.54 × 10^−6^ and *B* = 6.83 × 10^9^. Since it is difficult to know the actual surface field, *F* is usually replaced by *βE*, where *β* is the field enhancement factor and *E* is the applied field. Then, Equation (1) can be rewritten as

(2) $$J=\frac{A\beta^{2}E^{2}}{\phi \;}\exp\left(\frac{-B\phi^{3/2}}{\beta E}\right)$$

According to Equation (2), a simple criterion for the electron emission to obey the FN theory is to obtain a straight line in the FN plot with ln(*J*/*E*^2^) versus 1/*E*. Moreover, by fitting the slope of the FN plot *k_FN_* = −*B*/*β*, one can obtain the *β* of the cathode.

**Figure 15:**
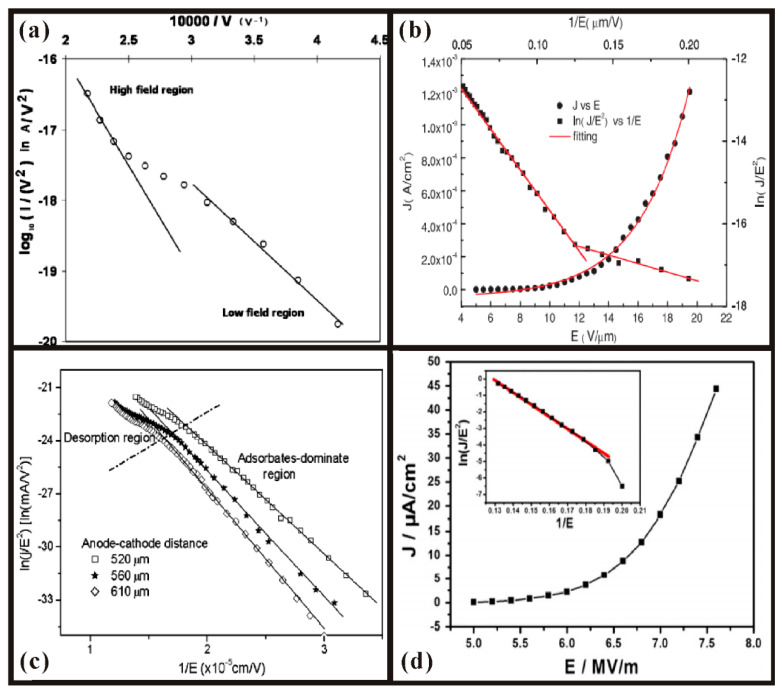
Some non-linear (**a**–**c**) and typical linear (**d**) Fowler–Nordheim (FN) plots in the field emission of ZnO nanowires. (**a**) Reproduced from [73], with the permission of ACS Publications, 2010. (**b**) Reproduced from [74], with the permission of IOP Publishing, 2007. (**c**) Reproduced from [75], with the permission of AIP Publishing, 2004. (**d**) Reproduced from [70], with the permission of IOP Publishing, 2014.

Although some references have reported the non-linear FN plots in the field emission of ZnO nano field emitters, which the authors attributed to the conduction band current saturation (Figure 15a) [73], the surface state (Figure 15b) [74] or the desorption (Figure 15c) [75], the results in most of the other works still followed a straight line in the FN plot (Figure 15d) [70]. Consequently, people usually used the FN formula to fit their results and a very high field enhancement factor (one order magnitude higher than the geometrical field enhancement factor) can be deduced.

J. Zeng et al. [76] first reported this unusual phenomenon and proposed a field-induced hot electron emission model to explain the results. In their model, they believed the emission electron can be accelerated within the penetration field near the surface of ZnO nanowire and become hot electron with an effective electron temperature *T_e_*. Since these hot electrons have a lower effective surface barrier height, the effective work function of a ZnO nanowire field emitter will be smaller than the value of 5.3 eV which is usually used in the fitting of *β*. As a result, the fitted *β* will be overestimated. In an analogy to the case of dielectric thin film, they assumed the penetration field within ZnO can be treated as a uniform field and the electron scattering is dominated by acoustic phonon scattering. Then, *T_e_* can be approximately described as,

(3) $$T_{e}=\sqrt{\frac{3\pi }{32}\;}\;\frac{\mu_{0}}{u}T\frac{F}{\varepsilon_{r}}$$

where *μ*_0_ is the electron mobility, *u* is the sound velocity, *T* is the temperature and *ε_r_* is the relative dielectric constant of ZnO. By replacing the term of temperature by *T_e_* in the formula of Murphy and Good [77], the field induced hot electron emission current density from ZnO nanowire can be deduced as:

(4) $$J\left(F,T\right)=\frac{4\pi mekT_{e}}{h^{3}}\;\int_{E_{c}}^{W_{l}}\ln(1+e^{\frac{E_{F}-W}{kT_{e}}}){\left[1+e^{\frac{8\pi {\left(2m{\left|W\right|}^{3}\right)}^{1/2}}{3heF}}\;v\left(y\right)\right]}^{-1}\;dW+\frac{4\pi mekT_{e}}{h^{3}}\;\int_{W_{l}}^{\infty }\ln(1+e^{\frac{E_{F}-W}{kT_{e}}})dW$$

where *k* is the Boltzmann constant, *h* is the Planck constant, *e* is the electron charge, *m* is the electron mass and *E_F_* is the Fermi level.

**Figure 16:**
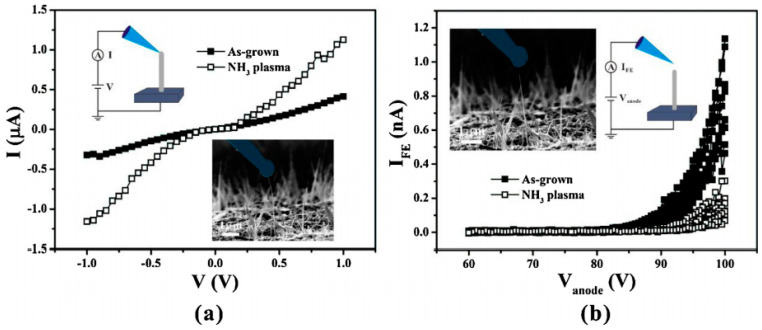
The electrical (**a**) and field emission (**b**) characteristics of an individual ZnO nanowire before and after the NH_3_ plasma treatment. Reproduced from [81], with the permission of Elsevier, 2018.

Considering that most of the reported carrier densities of ZnO nanowire are higher than 10^16^ cm^−3^ [78,79,80,81], the shielding effect of the high free electron density on the penetration field should be taken into account. This will invalidate the assumption for Zeng’s model that the penetration field is a uniform field and should cause an overestimation of *T_e_*. Y. Chen et al. [81] found evidences for this non-uniform penetration field from an in situ investigation on the electrical and field-emission characteristics from individual ZnO nanowire. By using NH_3_ plasma treatment, the conductance of ZnO nanowire can be modified with an unchanged morphology. Interestingly, they observed that ZnO nanowire with an enhanced conductance after NH_3_ plasma treatment had a worse field-emission property (Figure 16), which is in contradiction with the common understanding. To explain this discrepancy, they proposed a penetration length-dependent field-induced hot electron emission model. The basic idea for the model is that the length for the penetration field is finite which will vary with the carrier concentration of the nanowire. When the nanowire is in a higher conductance, the higher carrier density will screen the surface field which leads to a shorter penetration length. Since the hot electron energy is gained from the field acceleration within the penetration field, a shorter penetration length will result in an energetic lower hot electron, which reduces the field emission current in the low field section. However, when the emission current becomes larger at a higher field, the field-emission properties will be mainly dependent on the conductance which controls the electron supply. The mathematic formula of the model was subsequently given as [82]:

(5) $$J_{FHE}\left(F,T\right)=\frac{4\pi mekT_{e}}{h^{3}}\;C\int_{E_{c}}^{W_{l}}\ln(1+e^{\frac{E_{Fs}-W}{kT_{e}}}){\left[1+e^{\frac{8\pi {\left(2m{\left|W\right|}^{3}\right)}^{1/2}}{3heF}}\;v\left(y\right)\right]}^{-1}\;dW+\frac{4\pi mekT_{e}}{h^{3}}\;\int_{W_{l}}^{\infty }\ln(1+e^{\frac{E_{Fs}-W}{kT_{e}}})dW$$

where *C* is the ratio factor that balances the conductance in the nanowire body with that at its surface, and *T_e_* is rewritten as:

(6) $$T_{e}=\{\begin{array}{cc} T+\frac{2eV_{s}}{3k} & L_{D}\leq \lambda \\ T_{e0}+\frac{2}{3k}\int_{t_{0}}^{t}\left[\left(\frac{dE}{dt}\right)+\left(\frac{dE}{dt}\right)\right]dt & \lambda <L_{D} \end{array}$$

**Figure 17:**
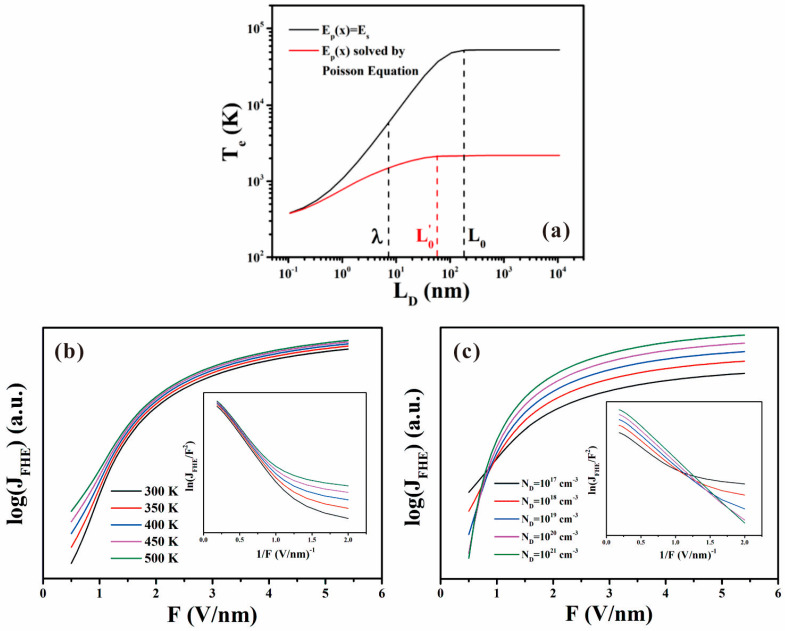
Calculation results based on penetration length-dependent hot electron emission model. (**a**) Simulated effective electron temperature as a function of the penetration length. (**b**) Simulated field emission characteristics under different temperature. (**c**) Simulated field emission characteristics with different carrier concentration. The insets in (**b**,**c**) are the corresponding FN plots. Reproduced from [82], with the permission of IOP Publishing, 2018.

Details for the formula deduction can be found in their work [82]. To have a better understanding, Figure 17a illustrated the comparison of *T_e_* calculated by using Zeng’s or Chen’s models. It is seen that the difference is more than one order of magnitude. By varying the temperature and carrier concentration, the field emission I–V characteristics and the corresponding FN plots can also be simulated as shown in Figure 17b,c. The positive and negative correlations between the emission current and the carrier density under strong and weak field in Figure 17c are consistent with the experimental results.

**Figure 18:**
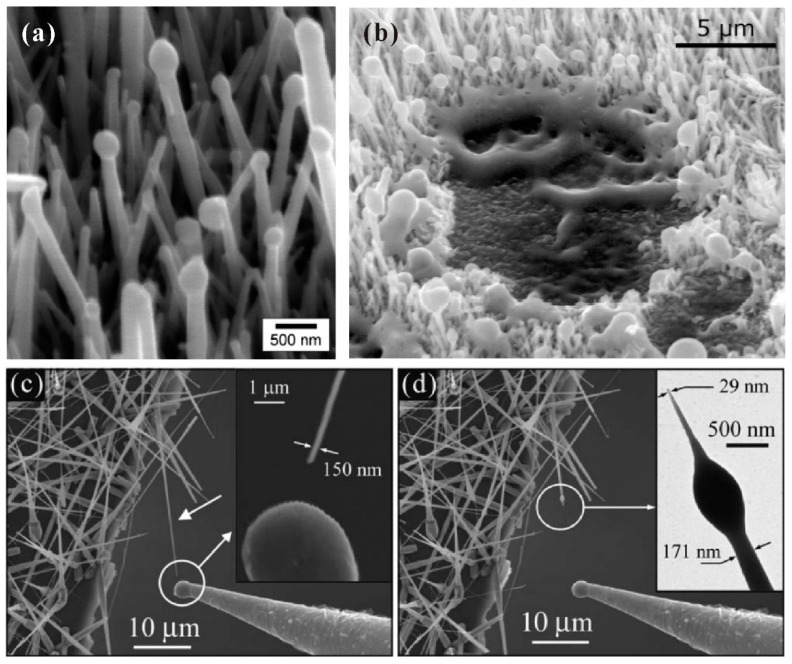
Joule heating induced vacuum breakdown in the field emission of ZnO nanowires. (**a**) The case when the applied field increased slowly. (**b**) The case when the applied field increased sharply. Reproduced from [53], with the permission of AIP Publishing, 2011. (**c**,**d**) The cases before and after vacuum breakdown for an individual nanowire. Reproduced from [65], with the permission of AIP Publishing, 2009.

Another important concern for the field-emission mechanism of ZnO nanowire is probably the joule heating-induced vacuum breakdown event, which is related to the maximum field emission current of ZnO nanowires and limits the applications. So far, many works have reported the melting phenomenon of a ZnO nanowire at its tip (Figure 18) when operating under large field-emission current [53,65,83]. To explain these findings, researchers have simulated the temperature at the nanowire’s tip under the operating current. For example, Z. Xiao et al. [65] calculated the tip temperature of ZnO nanowire can be as high as 2248 K (melting point for bulk ZnO). While in another work, V. Semet et al. [53] simulated the highest temperature at the nanowire’s tip should be lower than 1700 K, which they attributed to the nanowire having a lower melting point than its bulk value due to the size effect.

Since the positive feedback of joule heating on the emission current has not been considered in the above works, the questions as to whether ZnO nanowire can be steadily working with joule heating or not and how high the steady temperature can be remain unanswered. To answer these questions, Y. Chen et al. [60] investigated the steady state on the field emission of ZnO nanowire with joule heating by measuring the ring-shaped field-emission pattern. In an analogy to the work on CNT [84], the steady state is determined by both the thermal conduction along the nanowire and the thermal field-emission process. Considering that the thermal field emission current of ZnO nanowire can be described by Equation (5), the thermal conduction equation along the nanowire was given as:

(7) $$\pi r^{2}\kappa \frac{\partial^{2}T\left(x\right)}{\partial x^{2}}dx-2\pi rdx\sigma \left(T{\left(x\right)}^{4}-T_{0}^{4}\right)-E_{N}\frac{2\pi rJ_{FHE}\left(F\left(x\right),\;T\left(x\right)\right)dx}{e}+I{\left(x\right)}^{2}\rho \left(T\left(x\right)\right)dx/\pi r^{2}=0$$

**Figure 19:**
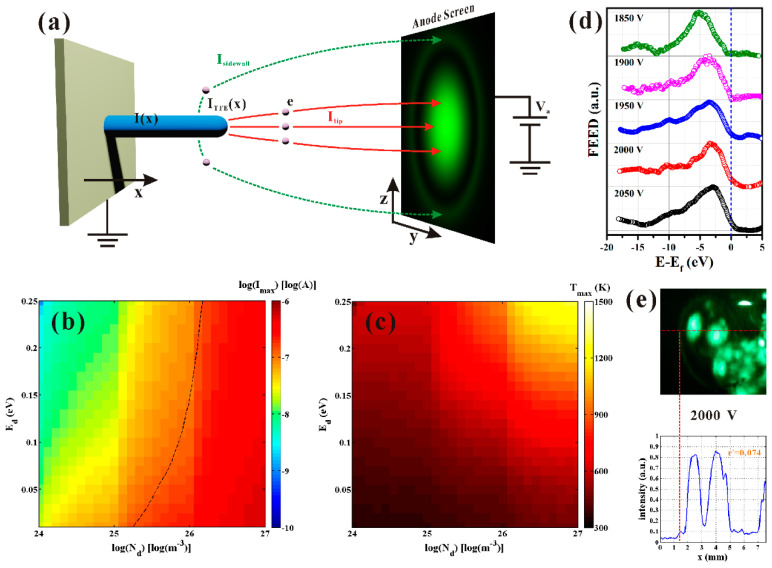
Field induced hot electron emission model of a ZnO nanowire under large current. (**a**) Schematic diagram of the emission current spatial distribution. (**b**) Calculated maximum emission current of individual ZnO nanowire with different electrical properties before thermal runaway. (**c**) Calculated maximum temperature of individual ZnO nanowire with different electrical properties before thermal runaway. (**d**) Experimental field emission energy distribution. (**e**) Experimental field emission ring pattern. Reproduced from [60], with the permission of John Wiley and Sons, 2020.

Here, the thermal field electron emission from the nanowire sidewall has been taken into account which the schematic diagram for the model can be seen in Figure 19a. Therefore, the third term on the left of Equation (7) is the energy flow caused by the Nottingham effect and the transportation current *I*(*x*) should have a distribution which can be expressed as:

(8) $$I\left(x\right)=I_{All}-2\pi r\overset{x}{\underset{0}{\int }}J_{FHE}\left(F\left(x\right),\;T\left(x\right)\right)dx$$

By solving Equations (5), (7) and (8) consistently, the steady current and temperature of ZnO nanowire with joule heating could be obtained depending on the field. When the field is strong enough, the current or temperature cannot reach a steady state and the nanowire will undergo a thermal runaway process. As the field decreases, a steady state can be obtained. If the steady temperature is higher than the melting point of the nanowire, the nanowire will be melted. Only when the steady temperature is lower than the melting point of nanowire, the calculated steady current and temperature are meaningful. The critical point whereby the nanowire will be melted or there will be thermal runaway is called the breakdown field. Under this framework, the maximum steady emission current and temperature before breakdown for an individual ZnO nanowire with different electrical properties (defect state density and excitation energy) were given as Figure 19b,c. The measured field-emission electron energy distribution and the field-emission ring-shaped pattern as shown in Figure 19d,e further verified their model, which indicated that the nanowire can be steadily heated to 900 K.

### 3.3. Field-Emission Optimization

Considering that the basic theory for the field-induced hot electron emission mechanism of ZnO nanowire is similar to the FN law except for the effective surface barrier height of the hot electron that is described by the effective electron temperature, the optimizations on its field emission properties can be carried out by modifying the geometrical structure, surface decoration and doping or back-contact resistance. Ideally, these three ways of modification respectively tune the field enhancement factor *β*, surface work function and electron supply. However, in an actual situation, their impacts can be much more complicated because the *β*, work function and electrical conductance are not independent on each modification. Another point worth noting is that although a lower carrier density will enhance the field-emission properties of ZnO nanowire at the small current stage as introduced above, its maximum field-emission current will still be limited by the electron supply at the large current stage, which requires high conductance. The details for each modification and their impacts will be covered in the following.

#### 3.3.1. Geometrical Structure

**Figure 20:**
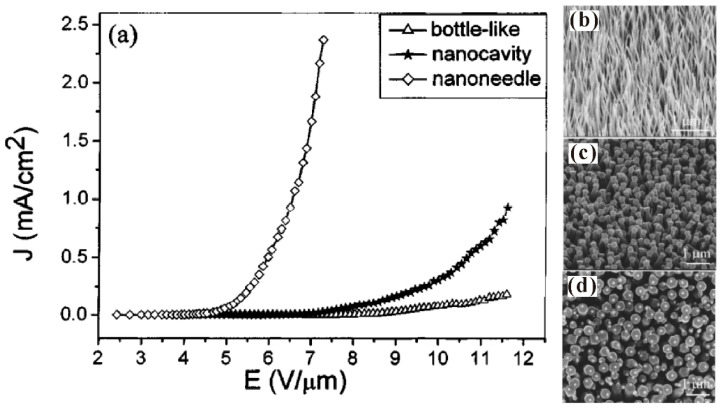
Influence of morphology on the field emission of ZnO nanorods. (**a**) Field-emission characteristics. (**b**–**d**) SEM images for ZnO nanorods with morphology of nanoneedle, nanocavity and bottle shaped. Reproduced from [85], with the permission of AIP Publishing, 2005.

**Figure 21:**
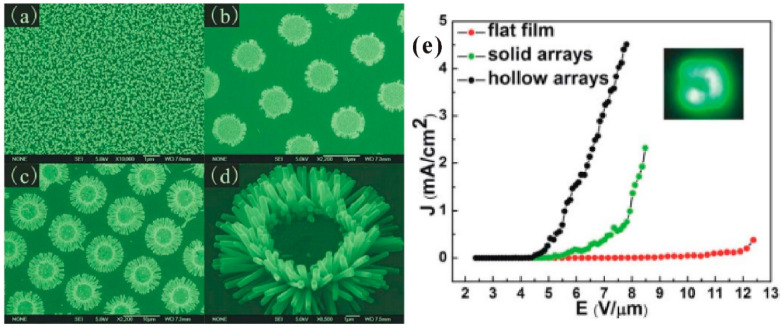
SEM images of ZnO nanorod with (**a**) flat film, (**b**) solid arrays and (**c**) hollow arrays. (**d**) The enlarged image of a single pattern in (**c**). (**e**) Field-emission properties of ZnO nanorod with different pattern structures. Reproduced from [90], with the permission of AIP Publishing, 2009.

**Figure 22:**
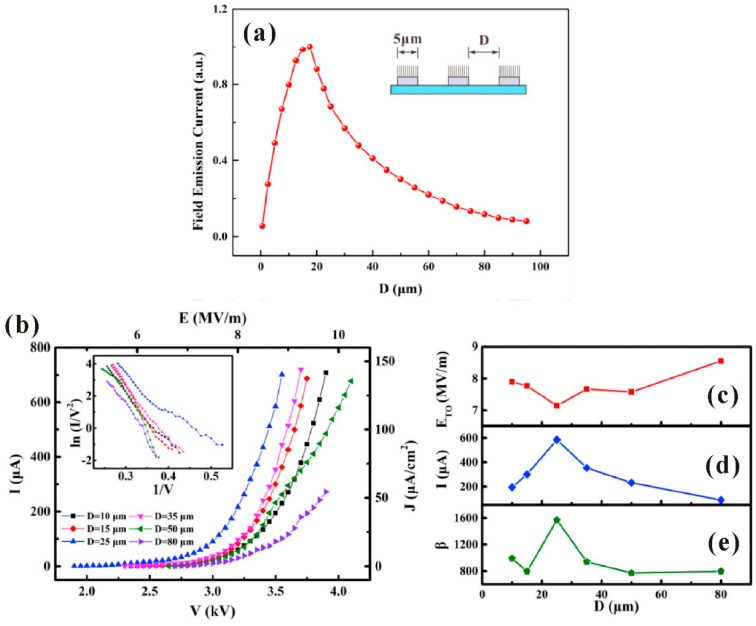
Field-emission properties of ZnO nanowires with different array spacing. (**a**) Simulated result. (**b**) Experimental result. The dependences of array spacing on the turn-on field (**c**), emission current (**d**) and the fitted *β* (**e**). Reproduced from [40], with the permission of Elsevier, 2019.

Theoretically, the modification of the geometrical structure and its impacts on the field-emission properties can be mainly divided into two aspects. One is for an individual nanowire that is to enhance its geometrical *β*. For example, Q. Zhao et al. [85] first investigated the morphology effects on the field emission of ZnO nanorod arrays. They found that the needle-like shaped nanorod has a lower turn-on field which is attributed to its higher value of *β* induced by the smaller tip radius (Figure 20). Similar results have also been reported by many other groups [86,87,88,89]. The other is for the nanowire arrays to avoid the screening effect. For example, N. Liu et al. [90] investigated the influence of pattern structure on the field emission of ZnO nanorod arrays. They proposed to use the pattern with a hollow center to fabricate ZnO nanorods at its edge (Figure 21c,d) rather than the flat film (Figure 21a) or solid pattern (Figure 21b), which can diminish the screening effect and reduce the turn-on field (Figure 21e). L. Wang et al. [40] studied the dependence of array spacing on the field-emission characteristics of a ZnO nanowire array. In their work, the field emission turn-on field of the nanowire arrays will firstly decrease then increase with the array spacing (Figure 22c). The emission current and the fitted *β* have opposite dependences (Figure 22d,e). Both the simulated (Figure 22a) and experimental (Figure 22b) results are consistent with each other.

However, in some cases, the situations are complicated due to the competition between the above two impacts. As an example, J. He et al. [91] synthesized four kinds of ZnO nanostructure and found that the best field-emission properties did not come from the sample with the sharpest tip structure. They attributed it to the fact that the sharpest tip structure samples tended to connect with each other, leading to intensive screening effect. Besides, other factors such as the electrical conductance variation could be involved. For instance, J. She et al. [67] investigated the field-emission characteristics of three kinds of ZnO nanostructure. They found that the agave-like structure has the lowest turn-on field and highest emission current, which is due to its better electrical conductance rather than the geometrical field enhancement factor.

#### 3.3.2. Surface Decoration

**Figure 23:**
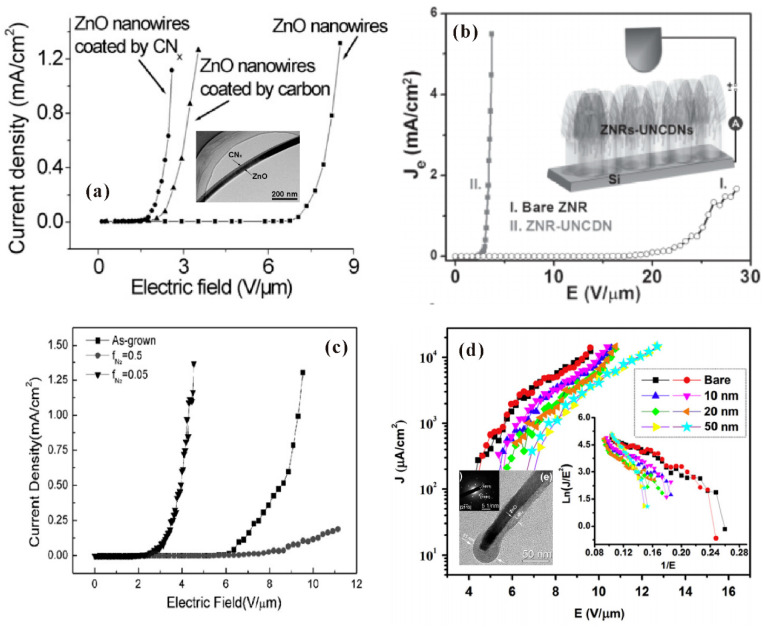
Field emission characteristics of ZnO nanostructures with or without coating. (**a**) Results for ZnO nanowires, ZnO nanowires coated with carbon or CNx. Reproduced from [92], with the permission of IOP Publishing, 2005. (**b**) Results for ZnO nanorods and ZnO nanorods coated with UNCDN. Reproduced from [93], with the permission of John Wiley and Sons, 2013. (**c**) Results for ZnO nanorods and HfN_x_ coated ZnO nanorods. Reproduced from [94], with the permission of Elsevier, 2008. (**d**) Results for bare and different thickness LaB_6_ coated ZnO nanowires. Reproduced from [95], with the permission of Elsevier, 2013.

A traditional way for surface decoration is to coat low work function materials on the cold cathode surface, which can lower the field-emission turn-on field. Considering that a ZnO nanowire is at the nanoscale, coating a thin film on it may also induce morphology changes which could influence the field-emission properties in a positive or negative way. For example, L. Liao et al. [92] prepared ZnO nanowires coated with an amorphous carbon or carbon nitride film and investigated their field-emission properties. Compared to the other samples, ZnO nanowires coated with a uniform carbon nitride film have lower turn-on field as shown in Figure 23a. This can be attributed to the reported low work function of carbon nitride film (0.01–0.1 eV) and the uniform coating thin film which has a negligible impact on the *β*. K. J. Sankaran et al. [93] fabricated ZnO nanorods coated with ultrananocrystalline diamond needles (UNCDN) and obtained enhanced field-emission characteristics as shown in Figure 23b. They attributed it to both the improvement of *β* and the reduction of work function. L. Yuan et al. [94] synthesized the HfN_x_ coated ZnO nanorods by using different N_2_ flow ratio (denoted as f_N2_ in Figure 23c). They found that the Hf_3_N_2_ coated sample synthesized by using f_N2_ of 0.05 has a lower turn-on field which was attributed to its low work function of 1.36 eV. However, the work by C.X. Zhao et al. [95] investigated the field emission of ZnO nanowires with coating of LaB_6_ thin film and found that the bare ZnO nanowires have the lower turn-on field. This can be understood as the coating increasing the apex radius of the nanowire, as shown in Figure 23d, which reduced the *β*.

**Figure 24:**
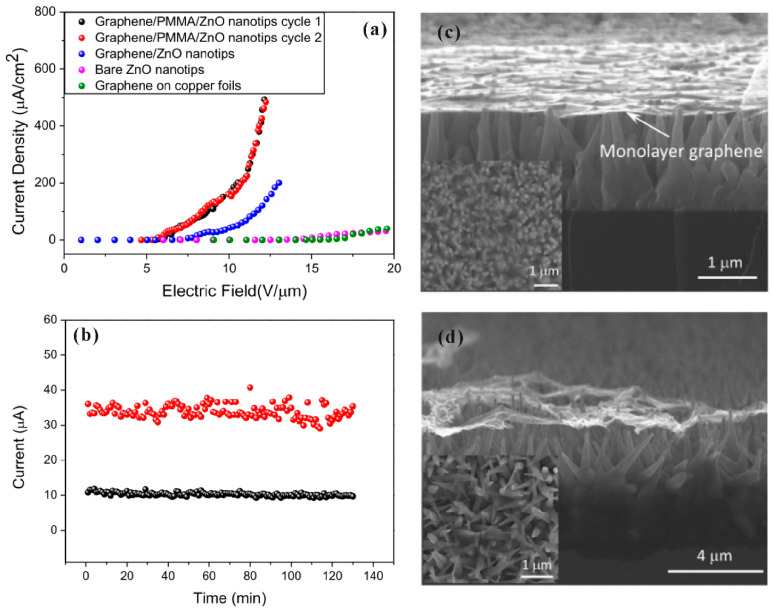
Field emission J-E curves (**a**) and stability (**b**) of the graphene/ZnO nanowires. The SEM images of the hybrid field emitter with (**c**) or without (**d**) using PMMA as the supporting layer. Reproduced from [97], with the permission of AIP Publishing, 2012.

Due to the unique properties and the dangling-bond-free lattice structure, stacking two-dimensional layered materials such as graphene and transition metal dichalcogenide (TMD) on ZnO nanowire has recently become an important means of surface decoration, which can lower the turn-on field and improve the stability. U.N. Maiti et al. [96] first fabricated graphene-ZnO nanowires by spin-coating the graphene dispersion on the nanowires substrate and obtained a reduced turn-on field. They believed the improved field-emission characteristics originated from the lower work function of graphene than ZnO and the multistage geometrical field enhancement at both the edge structure of graphene and the nanowire. Since it is difficult to fabricate monolayer graphene on ZnO nanowires by the spin-coating method, which can help to explore the intrinsic field-emission properties of this hybrid field emitter, Z. Yang et al. [97] proposed to utilize a spin-coated PMMA as the supporting layer and transfer monolayer graphene on it by using a stick-stamp method. After removing the PMMA, large area graphene with a flat surface is located on the ZnO nanowires which can be seen in Figure 24c. In comparison, the transferred graphene broke without using the PMMA, as shown in Figure 24d. From Figure 24a,b, it can be seen that the graphene-ZnO nanowire hybrid field emitter has a reduced turn-on field and an excellent stability, which was attributed to the localization at the nanotips of the hybrid emitter.

**Figure 25:**
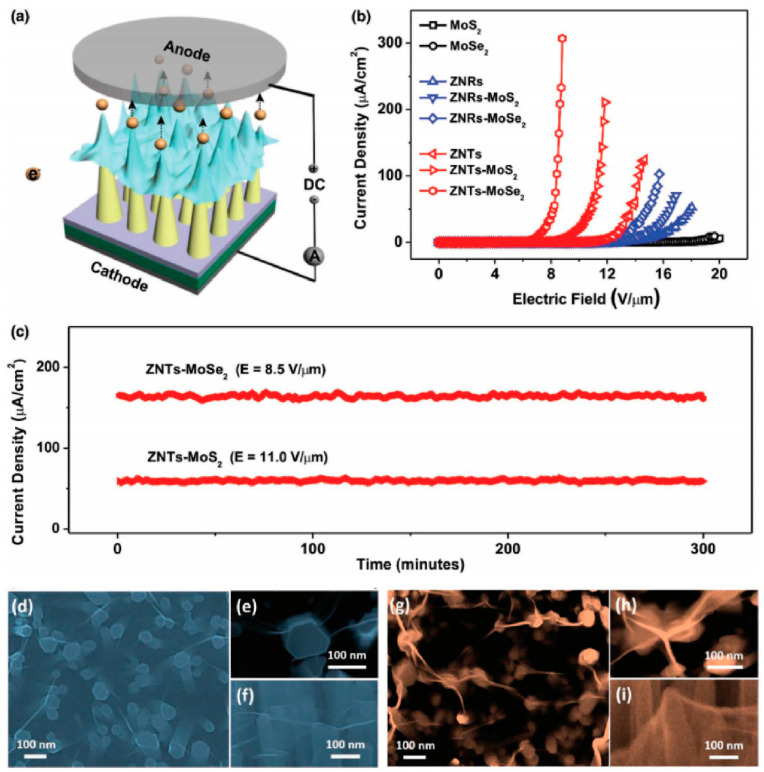
The measurement setup (**a**), field emission J-E curves (**b**) and stability (**c**) of the hybrid field emitters. The SEM images of the transition metal dichalcogenide (TMD) on ZnO nanorods (**d**–**f**) and TMD on ZnO nanotapers (**g**–**i**). Reproduced from [98], with the permission of John Wiley and Sons, 2017.

**Figure 26:**
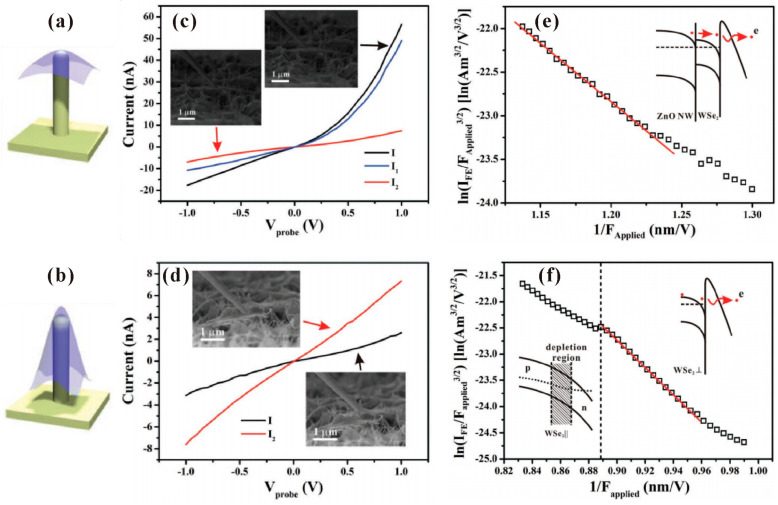
Schematic diagram for the “tip-contact” (**a**) and “edge-contact” (**b**) structures of WSe_2_-ZnO nanowire. Typical electrical characteristics of the “tip-contact” (**c**) and “edge-contact” (**d**) structures of WSe_2_-ZnO nanowire. Typical field emission FN plots of the “tip-contact” (**e**) and “edge-contact” (**f**) structures of WSe_2_-ZnO nanowire. Reproduced from [99], with the permission of John Wiley and Sons, 2019.

Apart from graphene, field-emission characteristics from TMDs on ZnO nanowires were also investigated. T.H Yang et al. [98] fabricated MoS_2_ or MoSe_2_ monolayers on different ZnO nanostructures and investigated the influence of morphology. Figure 25d–i show the SEM images of the MoSe_2_-ZnO nanorods (ZNRs) and MoSe_2_-nanotapers (ZNTs), respectively. It is seen that while the MoSe_2_-ZNR has a rippled morphology, the MoSe_2_-ZNT has a tent-like morphology. By using the diode measurement structure (Figure 25a), they found that sharper protrusions of the TMDs-ZNTs led to the enhanced field-emission characteristics when compared to the TMDs-ZNRs as shown in Figure 25b. As for the comparison between MoSe_2_-ZNT and MoS_2_-ZNT, the authors believed that this was attributed to the smaller band gap of MoSe_2_ which has a lower contact barrier between MoSe_2_ and ZnO. Highly stable field emission from MoS_2_-ZNTs and MoSe_2_-ZNTs were also demonstrated, as shown in Figure 25c. To further understand the mechanism of the morphology modulation on the field-emission properties of these TMDs-ZnO nanostructure hybrid emitters, Y. Chen et al. [99] investigated both the electrical and field-emission characteristics of an individual monolayer WSe_2_-ZnO nanowire emitter with different morphologies. Figure 26a,b show the schematic diagrams of two kinds of geometrical structures, which are referred to as the “tip-contact” and “edge-contact” structures. Their major difference is the curvature of WSe_2_ at the tip position. When in the “tip-contact” structure, the curvature of WSe_2_ is smaller than that of the supporting ZnO nanowire tip, which results that their contact is at the tip center of the nanowire cap. In the other case, their contact is at the edge of the nanowire cap and a part of WSe_2_ is suspended. By in situ measuring the electrical characteristics between the hybrid emitter tip or sidewall and the substrate for both the structures which can be seen in Figure 26c,d, they found that the electron transportation paths for the two structures are different. In the “tip-contact” case, the emission electron transports through the nanowire, its junction with WSe_2_, and WSe_2_ monolayer vertically. In the “edge-contact” case, the emission electron needs to transport an additional suspended part of WSe_2_ monolayer laterally. Hotter electrons can be formed in the former case which induced a lower turn-on field as can be seen in Figure 26e,f. Moreover, the lateral transportation of WSe_2_ led to a depletion layer during field emission, which caused the current saturation and the non-linear FN plots. In the “tip-contact” case, a high stability with current fluctuation of 0.79% was also obtained. Since the “tip-contact” structure only allows electron emission from the tip of the nanowire cap, it may be a possible way to reduce the emission spot of ZnO nanowire from its ring-shaped field emission pattern as shown in Figure 11. Further investigation on this field is expected.

#### 3.3.3. Doping and Back-Contact Resistance

**Figure 27:**
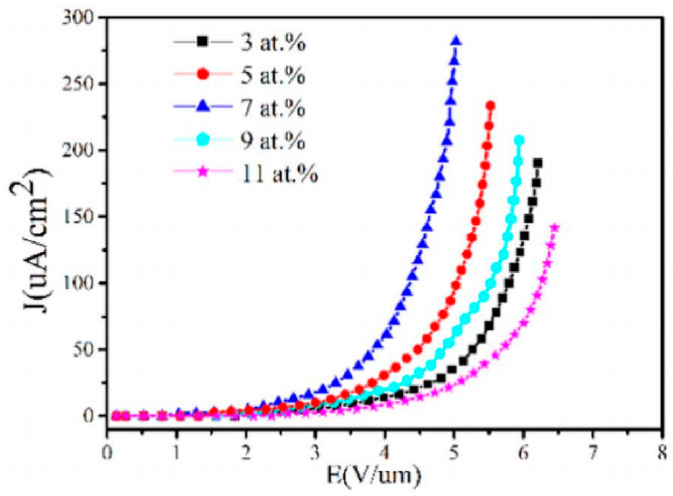
Field-emission characteristics of ZnO nanowires with Al doping concentration of 3, 5, 7, 9 and 11 at.%. Reproduced from [31], with the permission of Elsevier, 2018.

Generally, the modification on the doping can be carried out during the nanowire synthesis process, which can be found in Section 2. In many of the reported works, a reduced turn-on field and enhanced maximum current density can be obtained by n-type doping with Al [17], In [18] and Ge [20], which can be seen in Table 1. Since ZnO is a native n-type semiconductor, n-type doping can improve its electrical conductance, which accounts for its enhanced field emission properties. To further investigate the influence of doping concentration, Y. Lv et al. [31] fabricated Al-doped ZnO nanowires with doping concentrations of 3, 5, 7, 9 and 11 at.%. They found that as the doping concentration increases, the turn-on field will firstly reduce then increase, which has an optimal value when the doping concentration is 7 at.% (Figure 27). The underlying mechanism is believed to be related to the ZnO lattice distortion caused by the excessed Al impurities, which will capture the free electrons and reduce the electron supply. Moreover, the morphology effect on different doping ZnO nanowires may also contribute to this variation.

**Figure 28:**
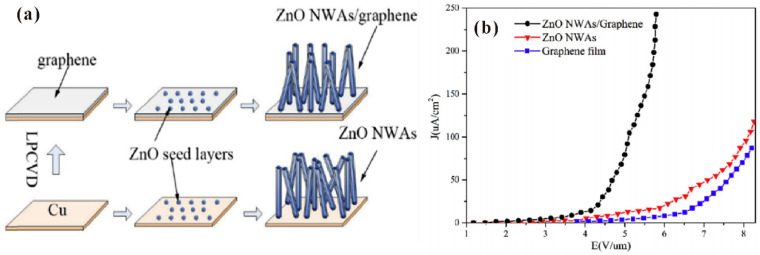
Fabrication procedures (**a**) and field-emission characteristics (**b**) of the ZnO nanowires grown on graphene layer. Reproduced from [102], with the permission of Elsevier, 2016.

In the early works on the modification of back-contact resistance, thin film layers such as AZO [46] and MgO [49] have been investigated as the buffer layer between ZnO nanowires and the substrate. Apart from the improved electron supply, the buffer layer can also adjust the morphology of the nanowire. As an example, Z.H. Chen et al. [46] fabricated ZnO nanowires with different morphology and density by tuning the thickness of the AZO buffer layer, the growth pressure and temperature. As introduced above, the reduced back-contact resistance can improve the emission current stability. Moreover, the turn-on field can be reduced and the maximum field-emission current density can increase as in the work of S. Chen et al. [49], which utilized an MgO buffer layer between the ZnO nanowires and substrate. Other work also utilized the band to band tunneling-induced thermo-enhancement between the ZnO nanowire and p-type Si to improve the field emission current [100]. Recently, the utilization of graphene as the buffer layer has been investigated due to its high mobility [101,102,103,104]. In a typical case, J. Liu et al. [102] fabricated ZnO nanowires on a graphene layer by using the hydrothermal method and obtained a reduced turn-on field. Figure 28 illustrates their fabrication procedures and the field-emission results.

## 4. Applications

The foremost motivation for the research on FEAs is probably to apply them in the flat panel field-emission display. Since other display techniques such as LCD, OLED and quantum dots have been chosen by the industry in recent years, this attempt has faded away and been transferred to the applications of flat panel light source, X-ray source and photodetector. In this section, the progress of the above applications based on ZnO nanowires cold cathode will be introduced in detail.

### 4.1. Field Emission Display (FED)

**Figure 29:**
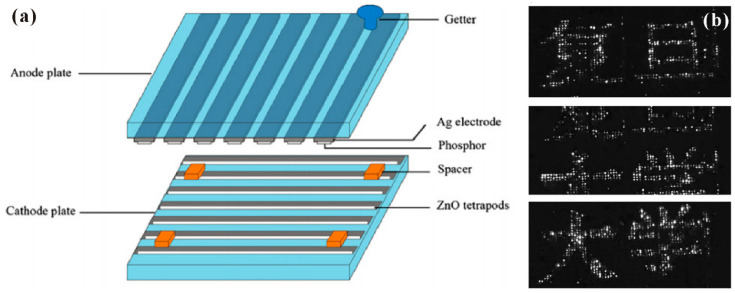
Demonstration of a diode structured field-emission display device based on ZnO nanotetrapods. (**a**) Device structure. (**b**) Some Chinese characters displayed by the device. Reproduced from [23], with the permission of Elsevier, 2008.

**Figure 30:**
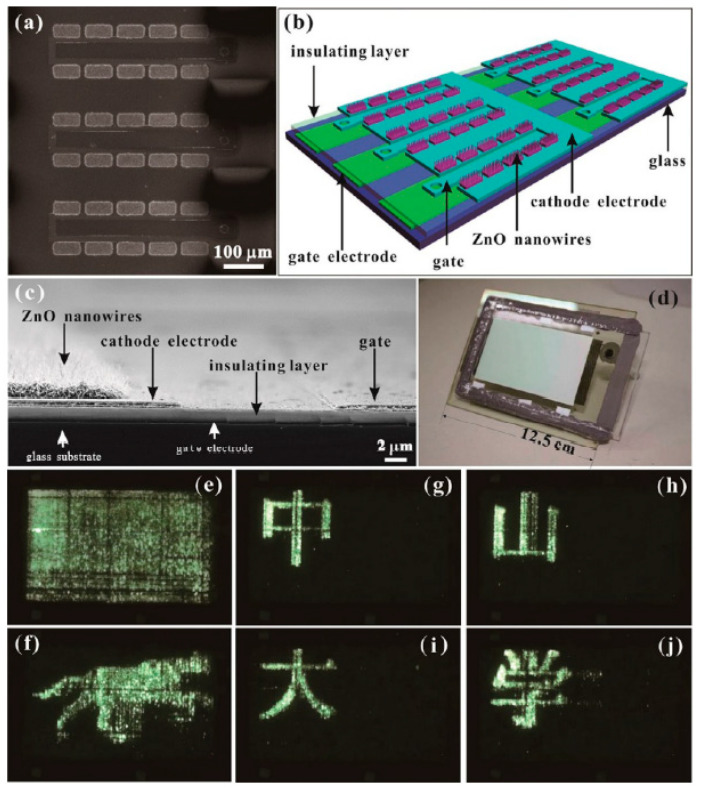
Demonstration of an addressable field emission device based on ZnO nanowires. Top view SEM image (**a**), schematic diagram (**b**), cross-sectional SEM image (**c**) and optical image (**d**) of the device. Full screen (**e**), cartoon of a running dog (**f**) and some Chinese characters (**g**–**j**) displayed by the device. Reproduced from [35], with the permission of ACS Publications, 2017.

Generally, there are two kinds of device structure that can realize field emitters with addressable function as required for FED. One is a diode structure whereby both the anode and cathode have electrode strips perpendicular to each other, the schematic diagram of which is shown in Figure 29a. When a high voltage is applied between one anode electrode strip and one cathode electrode strip, field emitters on the overlap region between the two electrodes can emit electrons, which works as one pixel of the device. By screen printing ZnO nanotetrapers on the cathode electrode of this kind of device structure, K. Zheng et al. [23] demonstrated the display of some Chinese characters as shown in Figure 29b. Another is a triode structure that consists of a phosphor-coated glass anode and the addressable gated FEAs, a typical gated structure of which fabricated by Y. Li et al. [35] can be seen in Figure 30a–d. In this device, the anode is applied with a fixed anode voltage. When a voltage is applied between the gate and cathode electrodes, the nanowires in the corresponding pixel can emit electrons. The display of full screen, cartoon image and some Chinese characters are demonstrated from the device, which are shown in Figure 30e–j.

**Figure 31:**
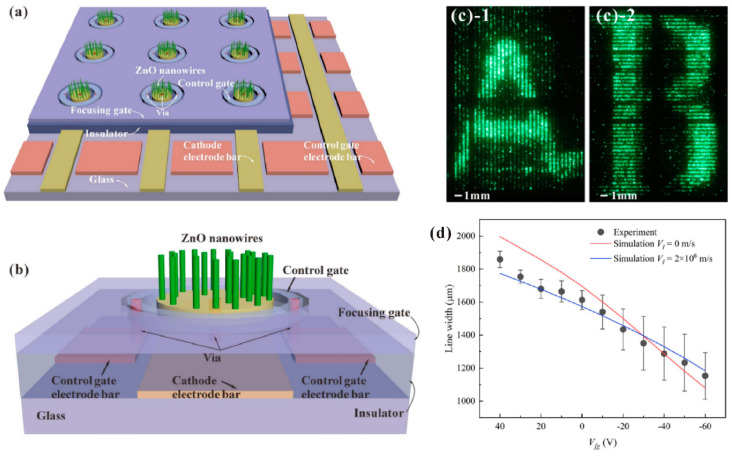
Demonstration of an addressable gated field-emission device with focusing gate based on ZnO nanowires. Schematic diagram for multi (**a**) and one (**b**) pixel of the device structure. English characters of “A” (**c-1**) and “B” (**c-2**) displayed by the device. (**d**) The relationship between the line width on the phosphor anode of one row pixels and the focusing gate voltage. Reproduced from [37], with the permission of IEEE, 2019.

To further meet the requirements of high-resolution device application, a focusing gate structure is needed for overcoming the problem of electron beam divergence. In view of this, Y. Liu et al. [105] demonstrated the ZnO nanowire FEAs with line-addressing and focusing capability. By changing the focusing gate voltage from 50 to −50 V, the linewidth of one row of the field-emission pattern can be reduced from 2.25 mm to 1.35 mm under a distance of 5 mm between the anode and cathode. X. Cao et al. [37] further upgraded this device with fully addressing capability by using a via structure, the schematic diagram of which can be seen in Figure 31a,b. The addressing capability of the device has been demonstrated by displaying some English characters as shown in Figure 31c, while the focusing ability still remains which can be seen in Figure 31d.

It should be pointed out that although FED no longer wins favor in the market of flat-panel displays, the techniques for field emitter arrays are still important for other flat-panel devices, such as UV illumination, X-ray source, e-beam lithography and photodetector, which are at their preliminary development stage.

### 4.2. Illumination

**Figure 32:**
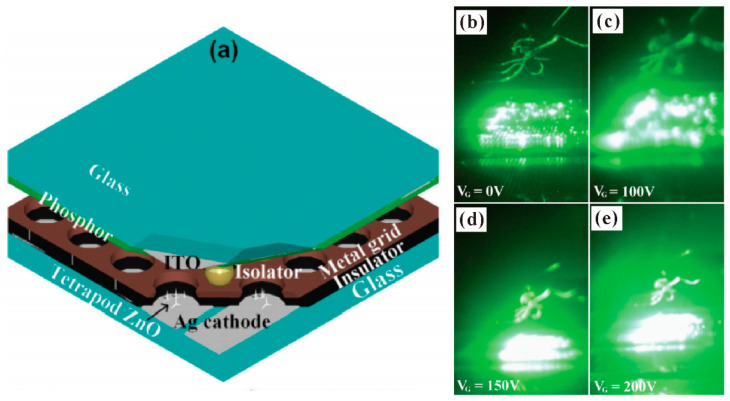
Demonstration of a field-emission light source based on ZnO nanostructures. (**a**) Schematic diagram of the device. (**b**–**e**) Field-emission images for gate voltage of 0, 100, 150 and 200 V. Reproduced from [106], with the permission of RSC Pub, 2014.

The principle for the field-emission light source is nearly the same as that for FED, which utilizes high-energy electrons to bombard the phosphor coated anode and produces cathodoluminescence. By contrast with the FED device, the addressable FEAs are not required for a light source, which makes it easier to be fabricated. For example, Y. Chen et al. [106] fabricated a triode structure field-emission light source based on ZnO nanotetrapods, the device structure of which can be seen schematically in Figure 32a. The gated controlled capability was demonstrated from the field-emission images recorded at different gate voltage (Figure 32b–e). By applying the anode voltage of 3300 V and gate voltage of 200 V, a luminous intensity of 8000 cd/m^2^ can be obtained from that device. Moreover, a diagonal 10-inch field emission light source with a diode structure is also fabricated by using ZnO nanowires [107].

**Figure 33:**
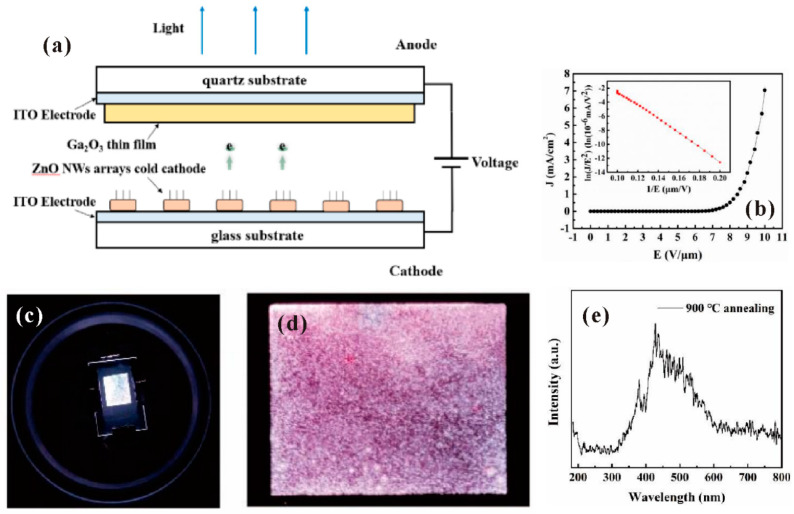
Demonstration of an ultraviolet (UV) light source based on ZnO nanowires cold cathode. (**a**) Schematic diagram of the device. (**b**) Field-emission characteristics. (**c**) Optical image of the operating device. (**d**) Emission pattern. (**e**) Cathodoluminescent spectrum. Reproduced from [108], with the permission of IEEE, 2020.

In addition to the visible light source, FEAs can also be utilized to generate the UV light by using the wide bandgap semiconductor as the anode. A field-emission UV source can avoid the problem of a solid-stated UV-LED that requires both n-type and p-type semiconductors for the radiation. This is because for some materials, e.g., ZnO, it is not so easy to realize stable doping of p-type impurities. A demonstration of a ZnO nanowires-based UV source has been reported by J. Yin et al. [108], the schematic diagram of the diode structure device of which is shown in Figure 33a. The field emission I-V characteristics and uniform field emission pattern of the device can be seen in Figure 33b–d. By using the polycrystalline Ga_2_O_3_ as the anode, the cathodoluminescent spectrum with peaks around 400 nm has been achieved, which is shown in Figure 33e.

### 4.3. Flat Panel X-ray Source

**Figure 34:**
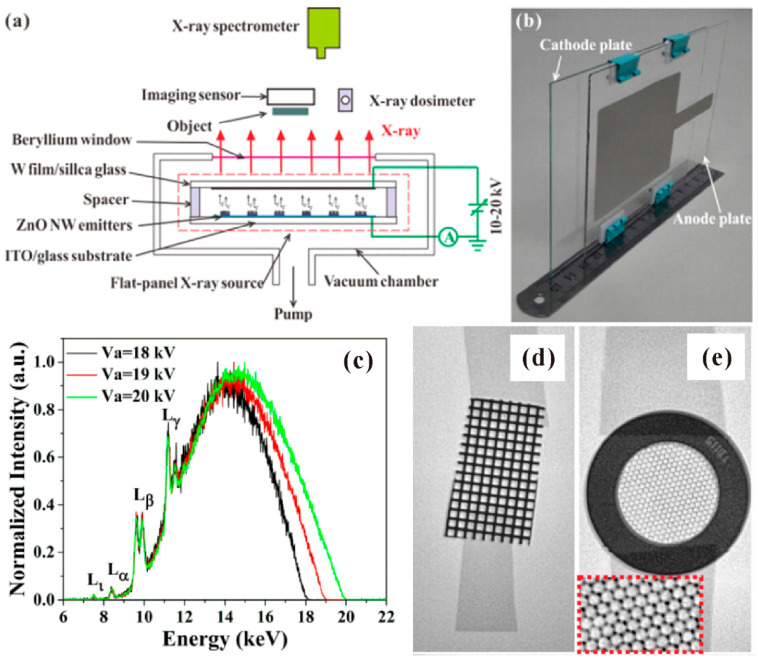
Flat-panel X-ray source based on ZnO nanowires. (**a**) Schematic diagram for the device structure. (**b**) Optical image of the device. (**c**) X-ray energy spectra. X-ray image of a metal mesh with linewidth of 200 μm (**d**) and 25 μm (**e**). Reproduced from [109], with the permission of AIP Publishing, 2015.

The flat panel X-ray source can be the next research spot to substitute the FED, because of the increasing demand for portable and low-cost medical X-ray computerized tomography (CT). Although the basic principle of this device is to use a metal target to replace the phosphor coated glass in the FED, a much higher anode voltage is needed for generating the X-ray radiation. On the other hand, whether the nanowire field emitter can sustain the X-ray irradiation is important for fabricating a reliable device. D. Chen et al. [109] first demonstrated a transmission-type flat-panel X-ray source using ZnO nanowires, the schematic diagram and optical image of which can be seen in Figure 34a,b. The X-ray energy spectra and the X-ray projected images of metal mesh with different linewidth can be seen in Figure 34c–e. Subsequently, a double-sided irradiating flat-panel X-ray source using ZnO nanowires was also demonstrated by their group [110], which utilized both the transmission and reflection of X-rays with ~200 mGy/min dose rate. Another flat-panel X-ray source has also been fabricated by using In-doped ZnO nanowires [41].

**Figure 35:**
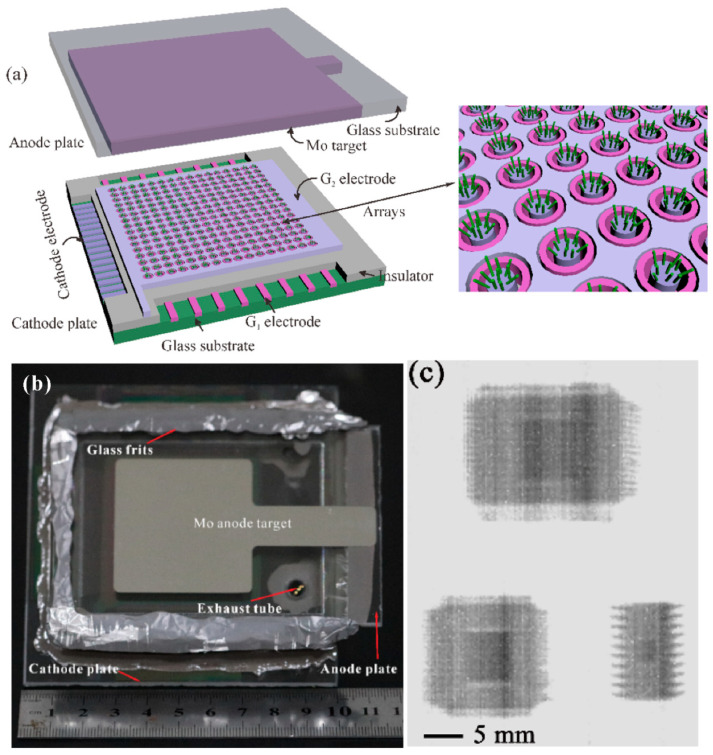
Addressable flat panel X-ray source based on ZnO nanowires. Schematic diagram (**a**) and optical image (**b**) of the device. (**c**) X-ray image of the integrated circuit chips. Reproduced from [111], with the permission of AIP Publishing, 2021.

Since the major advantage of a flat-panel X-ray source is its addressing capability, the ultimate goal for the research of flat-panel X-ray sources is to realize an addressable device. A successful demonstration of a fully vacuum-sealed addressable flat panel X-ray source based on ZnO nanowire FEAs has been reported recently by X. Cao et al. [111], the schematic diagram and optical image of which can be seen in Figure 35a,b. Although the X-ray image of the integrated circuit chips as shown in Figure 35c is blurred due to the low dose rate of ~200 nGy/s, their work opens up the possibility for realizing the functional addressable flat panel X-ray source, which could lead to a revolution in medical radiotherapy or CT systems.

**Figure 36:**
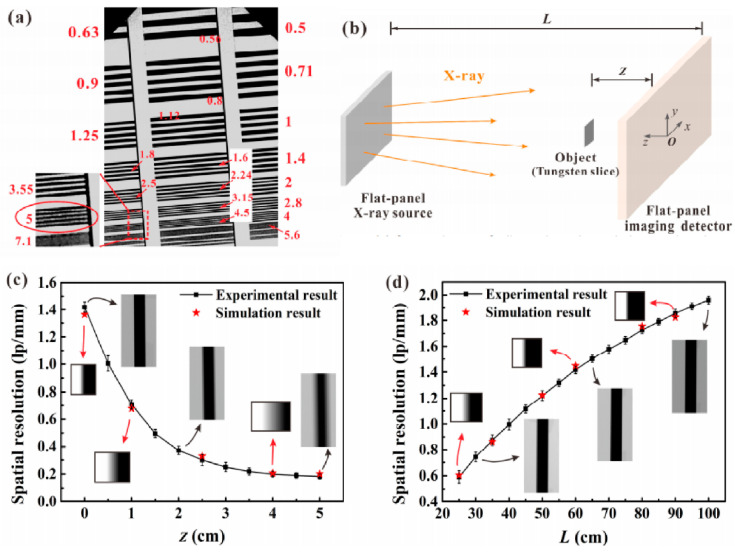
The imaging spatial resolution of a diagonal 4-inch X-ray source based on ZnO nanowires. (**a**) Contact image of a line-pair card. (**b**) Schematic diagram of the testing system. Dependence of spatial resolution on z (**c**) and L (**d**). Reproduced from [61], with the permission of IEEE, 2021.

Considering that the flat panel X-ray source has a larger radiant area than that of the usual X-ray tube, their performances in projection imaging should be different. To obtain a better understanding, L. Wang et al. [61] characterized the spatial resolution of a diagonal 4-inch flat panel X-ray source based on a ZnO nanowires cold cathode. Using the line-pair testing card, they obtained a contrast of more than 5 lp/mm (Figure 36a). To further investigate the dependence of object position on the resolution, they also used a modulation transfer function (MTF) analysis method to characterize the imaging spatial resolution, the measurement setup of which can be seen schematically in Figure 36b. The monotonic decrease and increased dependences of the distance between object and detector (z) and the distance between X-ray source and detector (L) on the resolution are found, which can be seen in Figure 36c,d. Since this result is based on a diode structure device, further investigations on the imaging performance of an addressable device are expected.

### 4.4. Photodetector

**Figure 37:**
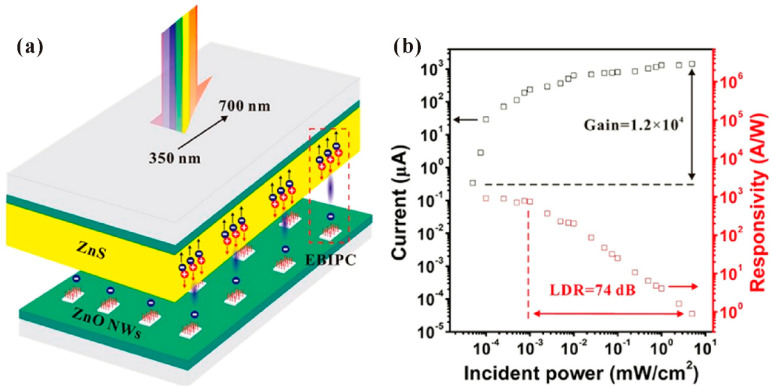
Demonstration of a photodetector based on a ZnO nanowire cold cathode. Schematic diagram (**a**) and performance (**b**) of the device. Reproduced from [112], with the permission of ACS Publications, 2018.

**Figure 38:**
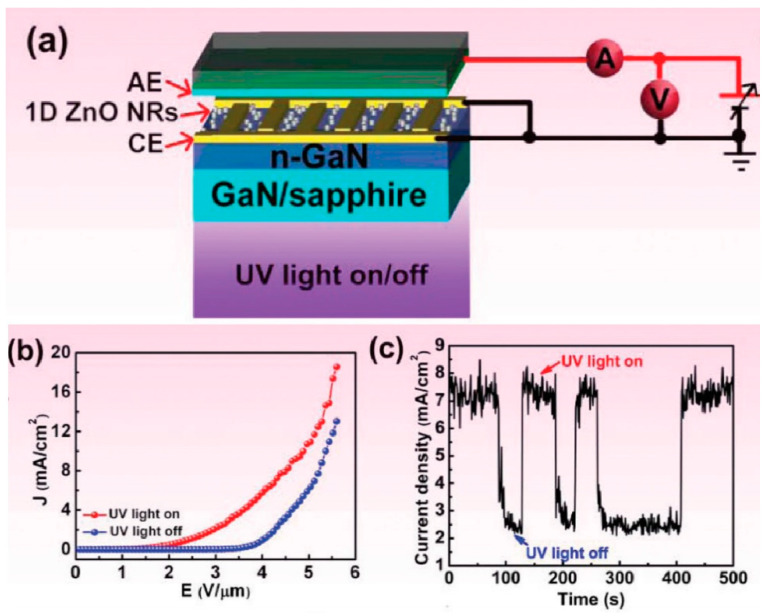
Schematic diagram (**a**), field-emission J-E characteristics (**b**) and time-resolved emission current density (**c**) of a ZnO nanorods/n-GaN photodector. Reproduced from [113], with the permission of RSC Pub, 2019.

Generally, there are two kinds of integrated photodetectors based on the ZnO nanowire cold cathode. One is to use a photoconductor material as the anode, the other is to fabricate a photoconductor material between the ZnO nanowires and the cathode electrode. Although both of them seem to work as a diode connecting with a photoconductor in series, their mechanisms are different. In the former case, as shown in Figure 37, Z. Zhang et al. [112] found that the electron bombardment on the ZnS photoconductor thin film can induce photoconductivity, which led to an internal photoconductive gain of above 10^4^. While in the latter case as shown in Figure 38, the emission current density of ZnO nanorod on n-GaN fabricated by Y. Chen et al. [113] can only be improved from <3 to 8 mA/cm^2^ under UV illumination. Although their attempt is to utilize the positive-feedback enhanced emission current to pump the AlGaN semiconductor for a UV light source, their device also has photodetection capability due to the enhanced electron supply for injection from GaN to the ZnO nanorod. Therefore, their work is presented here for a comparison.

In addition, a photodetector device based on a ZnO nanowires cold cathode has also been demonstrated for X-ray detection [114], in which a vacuum diode is connected with the photoconductor in series.

## 5. Perspectives

Although there has been an upsurge in research on cold cathode materials and applications in recent year, a practical cold cathode is still in great demand for overcoming the problems of thermionic cathode, e.g., bulky, high power consumption, low brightness, etc., which has always been a concern of the academic community and industry. Compared to an individual Spindt-type metal tip or CNT, a single ZnO nanowire has a much smaller maximum emission current and wider energy spread, which may not be suitable for the application of a point electron source. However, large-area ZnO nanowire FEAs can have high uniformity, low cost, good compatibility and stability, which exhibits great potential in large-area vacuum microelectronics devices, e.g., FED, flat-panel X-ray sources and detectors. Although the attempt to apply large-area FEAs in FED was given up about a decade ago, its application in flat-panel X-ray sources is promising, since there is no alternative technique at the present stage. To meet the requirement for a commercial product, further improvement for the performance of ZnO nanowire FEAs is needed. From the above comprehensive review, some future research trends on ZnO nanowire FEAs can be outlined as follows.

A larger emission current density for FEAs is always needed for practical use. For ZnO nanowire FEAs, there are two factors that limit the emission current density. One is its intrinsic field-emission current from individual nanowire, which is usually <1 μA. Considering that thermal runaway of ZnO nanowire is the major reason for its vacuum breakdown process, utilization of a pulsed electrical field to avoid the uncontrollable self-heating of nanowire should be a possible way to obtain a stable and enhanced transient emission current. Moreover, under the pulsed electrical field, phenomenon that combining the field emission process and the piezoelectric or pyroelectric effect of ZnO nanowire may also exist, which could lead to novel applications. The other factor is the screening effect, which causes the emission current density to decrease as the cathode area increases. Although some efforts have been made to avoid the screening effect by optimizing the cathode pattern structure and the pattern spacing, the state of the art of emission current density from ZnO nanowire FEAs is still not enough (<1 mA/cm^2^ for cathode area > 10 cm^2^). Further investigation should consider the space charge effect caused by the local emission hot spot. This is because the space charge of the local emission hot spot not only limits its emission current but also induces a screening effect on the surrounding nanowires. Research on the field-emission conditioning process may be useful to eliminate the emission hot spot.

For applications of ZnO nanowire FEAs, a highly convergent e-beam of each pixel is desired for high-resolution addressable capability, which is related to the device structure and the emission electron velocity distribution. The state of the art of the addressable ZnO nanowire FEAs uses an in-plane focusing structure to realize converged e-beam with ~1 mm linewidth. Since a gate electrode is located between the focusing electrode and the nanowire in an in-plane structure, its focusing capability should be weaker than that of the out-of-plane structure where the focusing electrode is located above the gate electrode. Future research on the out-of-plane structure based on ZnO nanowire FEAs is expected to obtain a more converged e-beam. So far, the emission electron velocity distribution can be reflected on the field emission microscopic pattern and field-emission energy distribution. From the ring-shaped field-emission pattern of ZnO nanowire, the initial velocity of the emission electron from the edge of the nanowire cap was estimated as ~10^6^ m/s. Moreover, field-emission energy distribution measurement of a ZnO nanowire indicated a wide energy spread of >3 eV. Both of these results can be attributed to its field-induced hot electron emission mechanism, which make it difficult to be converged. To overcome this problem, surface decoration using two-dimensional layered materials may be taken into account. Since the contact position between the nanowire and the 2D material determines the emission site, it can prevent electron emission from the edge of the nanowire cap which reduces the size of emission pattern.

## References

[1] Choi W.B., Chung D.S., Kang J.H., Kim H.Y., Jin Y.W., Han I.T., Lee Y.H., Jung J.E., Lee N.S., Park G.S. (1999). Fully sealed, high-brightness carbon-nanotube field-emission display. Appl. Phys. Lett..

[2] Teo K.B.K., Chhowalla M., Amaratunga G.A.J., Milne W.I., Legagneux P., Pirio G., Gangloff L., Pribat D., Semet V., Binh V.T. (2003). Fabrication and electrical characteristics of carbon nanotube-based microcathodes for use in a parallel electron-beam lithography system. J. Vac. Sci. Technol. B Microelectron. Nanometer Struct..

[3] Travish G., Rangel F.J., Evans M.A., Hollister B., Schmiedehausen K. Addressable flat-panel x-ray sources for medical, security, and industrial applications. Proceedings of the Advances in X-ray/EUV Optics and Components VII.

[4] Zhang Z., Wang K., Zheng K., Deng S., Xu N., Chen J. (2018). A flat panel photodetector formed by a ZnS photoconductor and ZnO nanowire field emitters achieving high responsivity from ultraviolet to visible light for indirect-conversion X-ray imaging. J. Light. Technol..

[5] Spindt C.A. (1968). A thin-film field-emission cathode. J. Appl. Phys..

[6] Zhang H., Tang J., Yuan J., Yamauchi Y., Suzuki T.T., Shinya N., Nakajima K., Qin L.-C. (2015). An ultrabright and monochromatic electron point source made of a LaB6 nanowire. Nat. Nanotechnol..

[7] Shen Y., Xu N., Deng S., Zhang Y., Liu F., Chen J. (2014). A Mo-nanoscrew formed by crystalline Mo-grains with high conductivity and excellent field emission properties. Nanoscale.

[8] Sui M., Gong P., Gu X. (2013). Review on one-dimensional ZnO nanostructures for electron field emitters. Front. Optoelectron..

[9] Young S.-J., Yang C.-C., Lai L.-T. (2017). Review—Growth of Al-, Ga-, and In-doped ZnO nanostructures via a low-temperature process and their application to field emission devices and ultraviolet photosensors. J. Electrochem. Soc..

[10] Wang Z.L. (2009). ZnO nanowire and nanobelt platform for nanotechnology. Mater. Sci. Eng. R Rep..

[11] Zhang Y., Ram M.K., Stefanakos E.K., Goswami D.Y. (2012). Synthesis, characterization, and applications of ZnO nanowires. J. Nanomater..

[12] Heo Y.W., Norton D.P., Tien L.C., Kwon Y., Kang B.S., Ren F., LaRoche J.R. (2004). ZnO nanowire growth and devices. Mater. Sci. Eng. Rep..

[13] Bagga S., Akhtar J., Mishra S. (2018). Synthesis and applications of ZnO nanowire: A review. AIP Conf. Proc..

[14] Li S., Zhang X., Yan B., Yu T. (2009). Growth mechanism and diameter control of well-aligned small-diameter ZnO nanowire arrays synthesized by a catalyst-free thermal evaporation method. Nanotechnology.

[15] Wang L., Zhang X., Zhao S., Zhou G., Zhou Y., Qi J. (2005). Synthesis of well-aligned ZnO nanowires by simple physical vapor deposition on c-oriented ZnO thin films without catalysts or additives. Appl. Phys. Lett..

[16] Ashraf S., Jones A.C., Bacsa J., Steiner A., Chalker P.R., Beahan P., Hindley S., Odedra R., Williams P.A., Heys P.N. (2011). MOCVD of vertically aligned ZnO nanowires using bidentate ether adducts of dimethylzinc. Chem. Vap. Depos..

[17] Zhang Z., Huang J., He H., Lin S., Tang H., Lu H., Ye Z. (2009). The influence of morphologies and doping of nanostructured ZnO on the field emission behaviors. Solid-State Electron..

[18] Ahmad M., Sun H., Zhu J. (2011). Enhanced photoluminescence and field-emission behavior of vertically well aligned arrays of in-doped ZnO nanowires. ACS Appl. Mater. Interfaces.

[19] Jia X., Xu H., Gao J., Jia X., Zhu H., Yu D. (2013). Ultralow electron mobility of an individual Cu-doped Zn O nanowire. Phys. Status Solidi.

[20] Liang Y. (2019). Ge-doped ZnO nanowire arrays as cold field emitters with excellent performance. Nanotechnology.

[21] Li S.Y., Lee C.Y., Lin P., Tseng T.Y. (2006). Gate-controlled ZnO nanowires for field-emission device application. J. Vac. Sci. Technol. Microelectron. Nanometer Struct..

[22] Ooki S., Ohshio S., Nishino J., Ohkawara Y., Ito H., Saitoh H. (2008). X-ray source with cold emitter fabricated using ZnO conductive whiskers. Jpn. J. Appl. Phys..

[23] Zheng K., Shen H., Li J., Sun D., Chen G., Hou K., Li C., Lei W. (2008). The fabrication and properties of field emission display based on ZnO tetrapod-liked nanostructure. Vacuum.

[24] Zhao Q., Huang C.-K., Zhu R., Xu J., Chen L., Yu D. (2011). 2D planar field emission devices based on individual ZnO nanowires. Solid State Commun..

[25] Kang H.W., Yeo J., Hwang J.O., Hong S., Lee P., Han S.Y., Lee J.H., Rho Y.S., Kim S.O., Ko S.H. (2011). Simple ZnO nanowires patterned growth by microcontact printing for high performance field emission device. J. Phys. Chem..

[26] Liu J., She J., Deng S., Chen J., Xu N. (2008). Ultrathin seed-layer for tuning density of ZnO nanowire arrays and their field emission characteristics. J. Phys. Chem..

[27] He H., She J.C., Huang Y.F., Deng S.Z., Xu N.S. (2012). Precisely-controlled fabrication of single ZnO nanoemitter arrays and their possible application in low energy parallel electron beam exposure. Nanoscale.

[28] Hsiao C.-H., Huang C.-S., Young S.-J., Chang S.-J., Guo J.-J., Liu C.-W., Yang T.-Y. (2013). Field-emission and photoelectrical characteristics of Ga–ZnO nanorods photodetector. Trans. Electron Devices.

[29] Mahmood K., Bin Park S., Sung H.J. (2013). Retracted article: Enhanced photoluminescence, raman spectra and field-emission behavior of indium-doped ZnO nanostructures. J. Mater. Chem..

[30] Shao D., Gao J., Xin G., Wang Y., Li L., Shi J., Lian J., Koratkar N., Sawyer S. (2015). Cl-doped ZnO nanowire arrays on 3D graphene foam with highly efficient field emission and photocatalytic properties. Small.

[31] Lv Y., Zhang Z., Yan J., Zhao W., Zhai C. (2018). Al doping influences on fabricating ZnO nanowire arrays: Enhanced field emission property. Ceram. Int..

[32] Ma T., Guo M., Zhang M., Zhang Y., Wang X. (2007). Density-controlled hydrothermal growth of well-aligned ZnO nanorod arrays. Nanotechnology.

[33] Lee C.Y., Li S.Y., Lin P., Tseng T.-Y. (2006). Field-emission triode of low-temperature synthesized ZnO nanowires. Trans. Nanotechnol..

[34] Zhao L., Chen Y., Liu Y., Zhang G., She J., Deng S., Xu N., Chen J. (2017). Integration of ZnO nanowires in gated field emitter arrays for large-area vacuum microelectronics applications. Curr. Appl. Phys..

[35] Li Y., Zhang Z., Zhang G., Zhao L., Deng S., Xu N., Chen J. (2017). Optimizing the field emission properties of ZnO nanowire arrays by precisely tuning the population density and application in large-area gated field emitter arrays. ACS Appl. Mater. Interfaces.

[36] Zhao L., Chen Y., Zhang Z., Cao X., Zhang G., She J., Deng S., Xu N., Chen J. (2018). Coplanar-gate ZnO nanowire field emitter arrays with enhanced gate-control performance using a ring-shaped cathode. Sci. Rep..

[37] Cao X., Yin J., Wang L., Zhang G., Deng S., She J., Xu N., Chen J. (2019). Fabrication of coaxis-gated ZnO nanowire field-emitter arrays with in-plane focusing gate electrode structure. Trans. Electron Devices.

[38] Zhao C.X., Li Y.F., Zhou J., Li L.Y., Deng S.Z., Xu N.S., Chen J. (2013). Large-scale synthesis of bicrystalline ZnO nanowire arrays by thermal oxidation of zinc film: Growth mechanism and high-performance field emission. Cryst. Growth Des..

[39] Zhang Z., Song X., Chen Y., She J., Deng S., Xu N., Chen J. (2017). Controllable preparation of 1-D and dendritic ZnO nanowires and their large area field-emission properties. J. Alloys Compd..

[40] Wang L., Zhao Y., Zheng K., She J., Deng S., Xu N., Chen J. (2019). Fabrication of large-area ZnO nanowire field emitter arrays by thermal oxidation for high-current application. Appl. Surf. Sci..

[41] Zhao Y., Chen Y., Zhang G., Zhan R., She J., Deng S., Chen J. (2021). High current field emission from large-area indium doped ZnO nanowire field emitter arrays for flat-panel X-ray source application. Nanomaterials.

[42] Lee C.J., Lee T.J., Lyu S.C., Zhang Y., Ruh H., Lee H.J. (2002). Field emission from well-aligned zinc oxide nanowires grown at low temperature. Appl. Phys. Lett..

[43] Wan Q., Yu K., Wang T.H., Lin C.L. (2003). Low-field electron emission from tetrapod-like ZnO nanostructures synthesized by rapid evaporation. Appl. Phys. Lett..

[44] Zhu Y.W., Zhang H.Z., Sun X.C., Feng S.Q., Xu J., Zhao Q., Yu D.P. (2003). Efficient field emission from ZnO nanoneedle arrays. Appl. Phys. Lett..

[45] Zhang Z., Yuan H., Zhou J., Liu N., Luo S., Miao Y., Gao Y., Wang J., Liu L., Song L. (2006). Growth mechanism, photoluminescence, and field-emission properties of ZnO nanoneedle arrays. J. Phys. Chem..

[46] Chen Z.H., Tang Y.B., Liu Y., Yuan G.D., Zhang W.F., Zapien J.A., Bello I., Zhang W.J., Lee C.-S., Lee S.T. (2009). ZnO nanowire arrays grown on Al: ZnO buffer layers and their enhanced electron field emission. J. Appl. Phys..

[47] Song J., Kulinich S.A., Yan J., Li Z., He J., Kan C., Zeng H. (2013). Epitaxial ZnO nanowire-on-nanoplate structures as efficient and transferable field emitters. Adv. Mater..

[48] Umar A., Algarni H., Kim S., Al-Assiri M.S. (2016). Time dependent growth of ZnO nanoflowers with enhanced field emission properties. Ceram. Int..

[49] Chen S., Chen J., Liu J., Qi J., Wang Y. (2016). Enhanced field emission from ZnO nanowire arrays utilizing MgO buffer between seed layer and silicon substrate. Appl. Surf. Sci..

[50] Zhang Z., Lv Y., Yan J., Hui D., Yun J., Zhai C., Zhao W. (2015). Uniform ZnO nanowire arrays: Hydrothermal synthesis, formation mechanism and field emission performance. J. Alloys Compd..

[51] Chen J., Lei W., Chai W., Zhang Z., Li C., Zhang X. (2008). High field emission enhancement of ZnO-nanorods via hydrothermal synthesis. Solid-State Electron..

[52] Wei A., Sun X.W., Xu C.X., Dong Z.L., Yu M.B., Huang W. (2006). Stable field emission from hydrothermally grown ZnO nanotubes. Appl. Phys. Lett..

[53] Semet V., Binh V.T., Pauporté T., Joulaud L., Vermersch F. (2011). Field emission behavior of vertically aligned ZnO nanowire planar cathodes. J. Appl. Phys..

[54] Hwang J.O., Lee D.H., Kim J.Y., Han T.H., Kim B.H., Park M., No K., Kim S.O. (2011). Vertical ZnO nanowires/graphene hybrids for transparent and flexible field emission. J. Mater. Chem..

[55] Zhang J., Su Y., Yang Z., Li M., Zhang Y. (2016). ZnO nanotapered arrays with successively modulated sharpness via a supersaturation-controlled hydrothermal reaction for efficient field emitters. Trans. Nanotechnol..

[56] Maiti U., Nandy S., Karan S., Mallik B., Chattopadhyay K. (2008). Enhanced optical and field emission properties of CTAB-assisted hydrothermal grown ZnO nanorods. Appl. Surf. Sci..

[57] Li X., Wang Y., Zhang Z., Ou H., She J., Deng S., Xu N., Chen J. (2018). Highly stable field emission from ZnO nanowire field emitters controlled by an amorphous indium-gallium-zinc-oxide thin film transistor. Jpn. J. Appl. Phys..

[58] Yang W., She J., Deng S., Xu N. (2012). Field emission from a MOSFET-controlled ZnO-nanowire cold cathode. Trans. Electron Devices.

[59] Li Q.H., Wan Q., Chen Y.J., Wang T.H., Jia H., Yu D.P. (2004). Stable field emission from tetrapod-like ZnO nanostructures. Appl. Phys. Lett..

[60] Chen Y., Luo S., Cao X., Li Y., She J., Deng S., Chen J. (2020). Stable heating above 900 K in the field emission of ZnO nanowires: Mechanism for achieving high current in large scale field emitter arrays. Adv. Electron. Mater..

[61] Wang L., Xu Y., Cao X., Huang J., Deng S., Xu N., Chen J. (2021). Diagonal 4-in ZnO nanowire cold cathode flat-panel X-ray source: Preparation and projection imaging properties. Trans. Nucl. Sci..

[62] Yeong K.S., Thong J.T. (2008). Field emission properties of individual zinc oxide nanowire field emitter. J. Vac. Sci. Technol..

[63] Huang Y., Zhang Y., Gu Y., Bai X., Qi J., Liao Q., Liu J. (2007). Field emission of a single in-doped ZnO nanowire. J. Phys. Chem..

[64] Dong L., Jiao J., Tuggle D.W., Petty J.M., Elliff S.A., Coulter M. (2003). ZnO nanowires formed on tungsten substrates and their electron field emission properties. Appl. Phys. Lett..

[65] Xiao Z.M., Li Z.B., Yang Y.H., Yang G.W., Deng S.Z., Xu N.S., She J.C., Chen J. (2009). Oscillating current observed in field emission from a single zinc oxide nanostructure and the physical mechanism. J. Appl. Phys..

[66] Huang Y., Bai X., Zhang Y., Qi J., Gu Y., Liao Q. (2007). Field-emission properties of individual ZnO nanowires studied in situ by transmission electron microscopy. J. Phys. Condens. Matter.

[67] She J., Xiao Z., Yang Y., Deng S., Chen J., Yang G., Xu N. (2008). Correlation between resistance and field emission performance of individual ZnO one-dimensional nanostructures. ACS Nano.

[68] Chen Y., Li Z., She J., Deng S., Xu N., Chen J. Field emission characteristics of individual ZnO nanowire before vacuum breakdown. Proceedings of the International Conference on Vacuum Nanoelectronics.

[69] Zhang X., Zhang G., Bai X., Zhao X., Xiao J., Wu Y., Lu F., Guo D. (2009). Field emission microscopy study of zinc oxide nanowires on tungsten tip. J. Vac. Sci. Technol..

[70] Chen Y.C., Deng S.Z., Xu N.S., Chen J. (2014). Origin of the ring-shaped emission pattern observed from the field emission of ZnO nanowire: Role of adsorbates and electron initial velocity. Mater. Res. Express.

[71] Al-Tabbakh A.A., More M.A., Joag D.S., Ramgir N.S., Mulla I.S., Pillai V.K. (2007). Energy analysis of field emitted electrons from a ZnO tetrapod. Appl. Phys. Lett..

[72] Fowler R.H., Nordheim L. Electron emission in intense electric fields. Proceedings of the Royal Society of London.

[73] Al-Tabbakh A.A., More M.A., Joag D.S., Mulla I.S., Pillai V.K. (2010). The fowler-nordheim plot behavior and mechanism of field electron emission from ZnO tetrapod structures. ACS Nano.

[74] Liu J.P., Xu C.X., Zhu G.P., Li X., Cui Y.P., Yang Y., Sun X. (2007). Hydrothermally grown ZnO nanorods on self-source substrate and their field emission. J. Phys. Appl. Phys..

[75] Zhang H.Z., Wang R.M., Zhu Y.W. (2004). Effect of adsorbates on field electron emission from ZnO nanoneedle arrays. J. Appl. Phys..

[76] Zeng J.Z., Deng S.Z., She J.C., He H., Xu N.S. (2010). Field-induced hot-electron emission model for wide-band-gap semiconductor nanostructures. J. Appl. Phys..

[77] Murphy E.L., Good R.H. (1956). Thermionic emission, field emission, and the transition region. Phys. Rev..

[78] Heo Y.W., Tien L.-C., Kwon Y., Norton D.P., Pearton S., Kang B.S., Ren F. (2004). Depletion-mode ZnO nanowire field-effect transistor. Appl. Phys. Lett..

[79] Yuan G.-D., Zhang W., Jie J., Fan X., Tang J.-X., Shafiq I., Ye Z.-Z., Lee C.-S., Lee S.-T. (2007). Tunable n-type conductivity and transport properties of Ga-doped ZnO nanowire arrays. Adv. Mater..

[80] Stiegler J., Tena-Zaera R., Idigoras O., Chuvilin A., Hillenbrand R. (2012). Correlative infrared-electron nanoscopy reveals the local structure–conductivity relationship in zinc oxide nanowires. Nat. Commun..

[81] Chen Y., Song X., Li Z., She J., Deng S., Xu N., Chen J. (2018). Penetration length-dependent hot electrons in the field emission from ZnO nanowires. Appl. Surf. Sci..

[82] Chen Y., Zhang Z., Li Z.-B., She J., Deng S., Xu N.-S., Chen J. (2018). Investigation of the temperature dependent field emission from individual ZnO nanowires for evidence of field-induced hot electrons emission. J. Phys. Condens. Matter.

[83] Dardona S., Peles A., Wrobel G., Piech M., Gao P.-X. (2010). Gas adsorption and high-emission current induced degradation of field emission characteristics in solution-processed ZnO nanoneedles. J. Appl. Phys..

[84] Huang N.Y., She J.C., Chen J., Deng S.Z., Xu N.S., Bishop H., Huq S.E., Wang L., Zhong D., Wang E.G. (2004). Mechanism responsible for initiating carbon nanotube vacuum breakdown. Phys. Rev. Lett..

[85] Zhao Q., Zhang H., Zhu Y.W., Feng S.Q., Sun X.C., Xu J., Yu D.P. (2005). Morphological effects on the field emission of ZnO nanorod arrays. Appl. Phys. Lett..

[86] Pan N., Xue H., Yu M., Cui X., Wang X., Hou J.G., Huang J., Deng S.Z. (2010). Tip-morphology-dependent field emission from ZnO nanorod arrays. Nanotechnology.

[87] Ye Z., Yang F., Lu Y., Zhi M., Tang H., Zhu L. (2007). ZnO nanorods with different morphologies and their field emission properties. Solid State Commun..

[88] Xiao J., Zhang X., Zhang G. (2008). Field emission from zinc oxide nanotowers: The role of the top morphology. Nanotechnology.

[89] Yang Y.H., Wang B., Xu N.S., Yang G.W. (2006). Field emission of one-dimensional micro- and nanostructures of zinc oxide. Appl. Phys. Lett..

[90] Liu N., Fang G., Zeng W., Long H., Yuan L., Zhao X. (2009). Diminish the screen effect in field emission via patterned and selective edge growth of ZnO nanorod arrays. Appl. Phys. Lett..

[91] He J., Zheng X., Hong X., Wang W., Cao Y., Chen T., Kong L., Wu Y., Wu Z., Kang J. (2018). Enhanced field emission of ZnO nanowire arrays by the control of their structures. Mater. Lett..

[92] Liao L., Li J.C., Wang D.F., Liu C.S., Fu Q., Fan L.X. (2005). Field emission property improvement of ZnO nanowires coated with amorphous carbon and carbon nitride films. Nanotechnology.

[93] Sankaran K.J., Afsal M., Lou S.-C., Chen H.-C., Chen C., Lee C.-Y., Chen L.-J., Tai N.-H., Lin I.-N. (2013). Electron field emission enhancement of vertically aligned ultrananocrystalline diamond-coated ZnO core-shell heterostructured nanorods. Small.

[94] Yuan L., Fang G., Li C., Li J., Wang M., Liu N., Zhao X. (2008). Field emission enhancement of ZnO nanorod arrays with hafnium nitride coating. Surf. Coat. Technol..

[95] Zhao C., Li Y., Chen J., Deng S., Xu N. (2013). Tunable field emission characteristics of ZnO nanowires coated with varied thickness of lanthanum boride thin films. Ultramicroscopy.

[96] Maiti U.N., Maiti S., Majumder T.P., Chattopadhyay K.K. (2011). Ultra-thin graphene edges at the nanowire tips: A cascade cold cathode with two-stage field amplification. Nanotechnology.

[97] Yang Z., Zhao Q., Ou Y., Wang W., Li H., Yu D. (2012). Enhanced field emission from large scale uniform monolayer graphene supported by well-aligned ZnO nanowire arrays. Appl. Phys..

[98] Yang T.-H., Chiu K.-C., Harn Y.-W., Chen H.-Y., Cai R.-F., Shyue J.-J., Lo S.-C., Wu J.-M., Lee Y.-H. (2017). Electron field emission of geometrically modulated monolayer semiconductors. Adv. Funct. Mater..

[99] Chen Y., Liu L., Zheng K., She J., Deng S., Xu N., Chen J. (2019). Highly stable field emission from a tungsten diselenide monolayer on zinc oxide nanowire by geometrically modulating hot electrons. Adv. Electron. Mater..

[100] Huang Z., Huang Y., Xu N., Chen J., She J., Deng S. (2018). Band-to-band tunneling-dominated thermo-enhanced field electron emission from p-Si/ZnO nanoemitters. ACS Appl. Mater. Interfaces.

[101] Song Z., Wei H., Liu Y., Wang J., Long H., Wang H., Qin P., Zeng W., Fang G. (2014). Enhanced field emission from aligned ZnO nanowires grown on a graphene layer with hydrothermal method. Trans. Nanotechnol..

[102] Liu J., Zhang Z., Lv Y., Yan J., Yun J., Zhao W., Kou L., Zhai C. (2016). Synthesis and characterization of ZnO NWAs/graphene composites for enhanced optical and field emission performances. Compos. Eng..

[103] Ding S., Zhou Y., Ye M., Lei W. (2017). Stable field emission from ZnO nanowires grown on 3D graphene foam. Vacuum.

[104] Wu J., Chen L., Li S., Du C., Zhang Q., Zheng C., Xu J., Song K. (2018). Improved field emission performances for graphene/ZnO nanowires/graphene sandwich composites. Mater. Lett..

[105] Liu Y., Zhao L., Zhang Z., Chen D., Zhang G., She J., Deng S., Xu N., Chen J. (2018). Fabrication of ZnO nanowire field-emitter arrays with focusing capability. Trans. Electron Devices.

[106] Chen Y., Hu L., Song H., Jiang H., Li D., Miao G., Li Z., Sun X., Zhang Z., Guo T. (2014). Optimized performances of tetrapod-like ZnO nanostructures for a triode structure field emission planar light source. Nanoscale.

[107] Xu J., Zhang Y., Deng S., Chen J., Xu N. 10 inch screen printed ZnO nanowire cold cathode for flat panel light source. Proceedings of the International Vacuum Nanoelectronics Conference.

[108] Yin J., Chen M., Wang L., Cao X., She J., Deng S., Xu N., Chen J. Cathodoluminescent properties of polycrystalline Ga_2_O_3_ thin film and its application UV flat panel light source. Proceedings of the 33rd International Vacuum Nanoelectronics Conference.

[109] Chen D., Song X., Zhang Z., Li Z., She J., Deng S., Chen J. (2015). Transmission type flat-panel X-ray source using ZnO nanowire field emitters. Appl. Phys. Lett..

[110] Chen D., Xu Y., Zhang G., Zhang Z., She J., Deng S., Chen J. (2017). A double-sided radiating flat-panel X-ray source using ZnO nanowire field emitters. Vacuum.

[111] Cao X., Zhang G., Zhao Y., Xu Y., She J., Deng S., Chen J. (2021). Fully vacuum-sealed addressable nanowire cold cathode flat-panel X-ray source. Appl. Phys. Lett..

[112] Zhang Z., Wang K., Zheng K., Deng S., Xu N., Chen J. (2018). Electron bombardment induced photoconductivity and high gain in a flat panel photodetector based on a ZnS photoconductor and ZnO nanowire field emitters. ACS Photonics.

[113] Chen Y., Zhang Z., Jiang H., Li Z., Miao G., Song H., Guo T. (2019). Realization of an efficient electron source by ultraviolet-light-assisted field emission from a one-dimensional ZnO nanorods/n-GaN heterostructure photoconductive detector. Nanoscale.

[114] Zhang Z., Zheng W., Wang K., Chen H., Deng S., Chen J. (2020). Sensitive and fast direct conversion x-ray detectors based on single-crystalline HgI2 photoconductor and ZnO nanowire vacuum diode. Adv. Mater. Technol..

